# Validating MEG estimated resting-state connectome with intracranial EEG

**DOI:** 10.1162/netn_a_00441

**Published:** 2025-03-20

**Authors:** Jawata Afnan, Zhengchen Cai, Jean-Marc Lina, Chifaou Abdallah, Giovanni Pellegrino, Giorgio Arcara, Hassan Khajehpour, Birgit Frauscher, Jean Gotman, Christophe Grova

**Affiliations:** Multimodal Functional Imaging Lab, Biomedical Engineering Department, McGill University, Montréal, Québec, H3A 2B4, Canada; Integrated Program in Neuroscience, McGill University, Montréal, Québec H3A 1A1, Canada; Montreal Neurological Institute, Department of Neurology and Neurosurgery, McGill University, Montréal, Québec H3A 2B4, Canada; Physnum Team, Centre De Recherches Mathématiques, Montréal, Québec, Canada; Electrical Engineering Department, École De Technologie Supérieure, Montréal, Québec H3C 1K3, Canada; Center for Advanced Research in Sleep Medicine, Sacré-Coeur Hospital, Montréal, Québec, Canada; Analytical Neurophysiology Lab, Department of Neurology, Duke University School of Medicine, Durham, North Carolina, USA; Epilepsy program, Schulich School of Medicine and Dentistry, Western University, London, Ontario N6A 5C1, Canada; Brain Imaging and Neural Dynamics Research Group, IRCCS San Camillo Hospital, Venice, Italy; Multimodal Functional Imaging Lab, Department of Physics and Concordia School of Health, Concordia University, Montréal, Québec, Canada

**Keywords:** MEG source imaging, Intracranial EEG, Connectivity, Source leakage, Resting state connectome

## Abstract

Magnetoencephalography (MEG) is widely used for studying resting-state brain connectivity. However, MEG source imaging is ill posed and has limited spatial resolution. This introduces source-leakage issues, making it challenging to interpret MEG-derived connectivity in resting states. To address this, we validated MEG-derived connectivity from 45 healthy participants using a normative intracranial EEG (iEEG) atlas. The MEG inverse problem was solved using the wavelet-maximum entropy on the mean method. We computed four connectivity metrics: amplitude envelope correlation (AEC), orthogonalized AEC (OAEC), phase locking value (PLV), and weighted-phase lag index (wPLI). We compared spatial correlation between MEG and iEEG connectomes across standard canonical frequency bands. We found moderate spatial correlations between MEG and iEEG connectomes for AEC and PLV. However, when considering metrics that correct/remove zero-lag connectivity (OAEC/wPLI), the spatial correlation between MEG and iEEG connectomes decreased. MEG exhibited higher zero-lag connectivity compared with iEEG. The correlations between MEG and iEEG connectomes suggest that relevant connectivity patterns can be recovered from MEG. However, since these correlations are moderate/low, MEG connectivity results should be interpreted with caution. Metrics that correct for zero-lag connectivity show decreased correlations, highlighting a trade-off; while MEG may capture more connectivity due to source-leakage, removing zero-lag connectivity can eliminate true connections.

## Author Summary

The ill-posed nature and low spatial resolution of EEG/magnetoencephalography (MEG) source imaging affects functional connectivity estimates, which become more complicated in the resting state due to the low signal-to-noise ratio. Several connectivity metrics have been proposed to address source leakage by removing zero-lag connectivity, although this can eliminate true neuronal zero-lag connections. Intracranial EEG (iEEG) is the gold standard for validating noninvasive measurements. In this study, we validated MEG-estimated connectivity for healthy subjects using the iEEG atlas of normal brain activity (Frauscher et al., 2018) as ground truth at a group level. We employed two amplitude-based metrics and two phase-based metrics. Our findings highlight how MEG connectivity compares with the iEEG atlas and provide valuable insights for resting-state EEG/MEG connectomic studies, particularly in the choice of connectivity metrics.

## INTRODUCTION

The study of brain connectomes is a rapidly growing field in neuroscience, which explores both the structural and functional patterns of resting-state brain connectivity, whereas electrophysiology plays a key role in disentangling static versus dynamic aspects of resting-state functional connectivity (Sadaghiani et al., 2022). Historically, MRI has been widely employed to investigate brain connectomes, encompassing structural connectivity assessed through diffusion MRI and functional connectome evaluated using functional MRI (fMRI). In contrast, the utilization of electrophysiological methods, such as noninvasive EEG/magnetoencephalography (MEG), in connectome research has experienced a notable surge in recent years. Due to their high temporal resolution and accessibility, EEG/MEG-based connectome studies have been undertaken to address a broad spectrum of questions in physiological and pathological conditions (Aydin et al., 2020; Xie & He, 2012). However, the main limitation of EEG/MEG-based connectome studies is that, as they involve scalp recordings and source localization, they require solving an ill-posed inverse problem (Darvas et al., 2004) and are therefore susceptible to source leakage. Source leakage, defined as the influence of a source on the estimation of the generators within its neighborhood (Brookes et al., 2012; Hedrich et al., 2017), is a significant concern, particularly for resting-state activity due to its low signal-to-noise ratio (SNR) condition. This affects the spatial accuracy of EEG/MEG estimated sources and introduces false positives in connectivity measures. Additionally, the use of connectivity measures that are insensitive to true near-zero-lag synchronization leads to false negatives (Palva et al., 2018; Palva & Palva, 2012). Validation is thus essential for noninvasive EEG/MEG resting-state source imaging techniques to ensure appropriate interpretation of connectome results.

Researchers have investigated EEG/MEG connectivity for resting-state activity, using simulations to study source leakage (Palva et al., 2018) or to assess the effect of source imaging parameters or the choice of regions of interest (ROIs) extraction on connectivity (Brkić et al., 2023; Hincapié et al., 2016; Vallarino et al., 2023), or when comparing networks derived from EEG/MEG sources with those from fMRI (Brookes et al., 2011a, 2011b; Rizkallah et al., 2020). Recently, a few studies have compared whole-brain EEG connectomes with fMRI-derived ones and found moderate spatial correlations between the two modalities in canonical frequency bands (Wirsich et al., 2017, 2020, 2021). However, because these modalities capture different brain mechanisms, electrophysiology in EEG versus hemodynamic activity in fMRI, direct comparisons are limited, particularly for specific frequency bands.

Compared with EEG/MEG, intracranial EEG (iEEG), commonly used in epilepsy presurgical evaluation, offers highly accurate estimation of brain activity with excellent spatial and temporal resolution, including good SNR from deep structures. iEEG measurements are also negligibly affected by volume conduction (Arnulfo et al., 2015; O’Reilly & Elsabbagh, 2021). However, it requires an invasive implantation procedure and has intrinsically limited spatial coverage, targeting only suspected regions of abnormal epileptic activity (Jayakar et al., 2016). Simultaneously recording EEG/MEG and iEEG provides probably the most reliable validation for noninvasive measurements (Koessler et al., 2010; Pizzo et al., 2019), as both modalities capture the same brain activity at the same temporal scale. However, validating whole-brain connectome estimates from EEG/MEG with iEEG is not feasible, as iEEG implantation covers the brain only partially. Therefore, validation is limited to the implanted brain regions only (Nir et al., 2008).

In this context, the iEEG atlas of resting-state human activity developed by Frauscher et al. (2018) at the Montreal Neurological Institute (MNI; https://mni-open-ieegatlas.research.mcgill.ca/) offers a unique opportunity for validating whole-brain connectome estimates from noninvasive EEG/MEG at the group level. This atlas pools data from many patients with epilepsy monitored during presurgical evaluation, retaining only iEEG electrodes implanted in healthy regions, that is, regions not exhibiting any epileptic discharges. We have successfully used this atlas to validate how the power spectra of resting-state oscillations could accurately be localized using MEG (Afnan et al., 2023). In this study, we propose a similar methodology to validate the resting-state connectome estimated from MEG within a group of healthy participants against the resting-state iEEG connectome derived from the MNI iEEG atlas. To our knowledge, this is the first study to compare cross-modal correlations between MEG and iEEG at a group level to validate MEG-derived connectivity in widespread brain regions. Even though MEG and iEEG data were not recorded simultaneously, they both represent connectivity of the healthy adult brain and should ideally be strongly correlated.

## MATERIALS AND METHODS

### Experimental Design

The iEEG connectome was constructed from the MNI iEEG atlas (110 subjects) of resting-state data (Ground Truth: iEEG Atlas). For MEG, resting-state data were obtained from 45 healthy subjects (Pellegrino et al., 2022). Wavelet-based maximum entropy on the mean (wMEM) was applied to solve the MEG inverse problem (MEG source imaging using wMEM; Afnan et al., 2023; Lina et al., 2014). MEG was reconstructed on subject-specific cortical surfaces and then projected to the positions of iEEG electrodes specified in the MNI iEEG atlas, using a method proposed by Grova et al. (2016; Estimation of virtual iEEG data from the MEG source map). Projecting the MEG source maps to the intracranial space facilitated a quantitative comparison between MEG and iEEG (Estimation of virtual iEEG data from the MEG source map). MEG connectomes were constructed using a bootstrapping approach described in Construction of reliable MEG connectomes using bootstrap resampling. Finally, we quantified the cross-modal spatial correlations between these two connectomes for six frequency bands: delta (0.5–4 Hz), theta (4–8 Hz), alpha (8–13 Hz), beta (13–30 Hz), low gamma (30–55 Hz), and high gamma (55–80 Hz). For each frequency band, the connectomes were constructed for four connectivity metrics: amplitude envelope correlation (AEC), AEC after pairwise orthogonalization (orthogonalized AEC [OAEC]; Hipp et al., 2012), phase locking value (PLV), and a modified version of weighted phase lag index (wPLI; Vinck et al., 2011), keeping only phase information (Estimation of Connectivity Metrics). We will denote the “MNI iEEG atlas” as the “iEEG atlas” for the remainder of the article.

### Ground Truth: iEEG Atlas

The iEEG atlas (Frauscher et al., 2018) was developed from 110 patients (age: 31 ± 10 years, range: 13–62 years, male [M]: 54) with refractory epilepsy who underwent iEEG implantation for presurgical epilepsy evaluation. The number of patients in the original paper was 106. However, by the time we started our project, additional patient data had been added. The atlas included electrodes in confirmed healthy brain regions, that is, channels that did not exhibit any epileptic discharges. It comprises 1,712 channels in a bipolar configuration. Each of the 1,712 channels has 60 s of resting-state data, recorded with eyes closed (sampling rate: 200 Hz). Preprocessing of iEEG data included filtering within the 0.5- to 80-Hz band and applying a notch filter at 50 Hz and 60 Hz to remove the line noise considering in which center (North America or Europe) the data were acquired. iEEG data were downsampled to 200 Hz if the original sampling rate was higher (original sampling rates were 200, 256, 512, 1,000, 1,024, and 2,000 Hz). The 60-s data were selected visually (either continuous or consecutive discontinuous > 5-s segments after artifact exclusion; Frauscher et al., 2018). The iEEG channels were grouped into 76 ROIs based on the Medical Image Computing and Computer-Assisted Intervention (MICCAI) atlas (38 ROIs in each hemisphere; Landman & Warfield, 2012). More details can be found in Frauscher et al. (2018).

#### Construction of iEEG connectome.

To compute connectivity between two ROIs in the iEEG atlas, it is necessary to have at least one pair of channels connecting them in the same subject (connectivity cannot be computed for pairs of ROIs recorded in different subjects). We identified all pairs of ROIs that exhibited at least one pair of channels between them. All local connections within the same ROI were discarded from further analysis. The number of channels between the ROI pairs and the number of subjects contributing to each ROI pair varied. For instance, some ROI pairs featured one or more pairs of channels from a single subject, while others could be contributed by up to 10 subjects, each providing one or more pairs of channels. Therefore, the average number of channels in all ROI pairs was 14 ranging from 1 to 217 channel pairs. For each pair of channels (between ROIs), connectivity was calculated using the four connectivity metrics described in Estimation of Connectivity Metrics. The connectivity values for each ROI pair were then averaged, irrespective of whether they belonged to the same or different subjects, resulting in a single connectivity value per ROI pair. This process resulted in a connectome covering 44% of the whole connectome, consisting of 1,278 pairs of ROIs, involving 100% of the MICCAI atlas (i.e., all 76 ROIs). Most connections were intrahemispheric, covering 62% of the left hemispheric connectome, 59% of the right hemispheric connectome, and 28% of interhemispheric connectome (see Supporting Information Figure S1 for more details).

### MEG

This study included 57 healthy participants who underwent MEG acquisition (resting state, with eyes closed), collected at the MEGLab of the Istituto di Ricovero e Cura a Carattere Scientifico San Camillo Hospital in Venice, Italy (Pellegrino et al., 2022). MEG was acquired using a CTF-MEG system (VSM MedTech Systems Inc., Coquitlam, BC, Canada) with 275 axial gradiometers with a sampling rate of 1,200 Hz. MEG preprocessing was performed with Brainstorm software (Tadel et al., 2011). Preprocessing of MEG data included (a) filtering within the 0.5- to 80-Hz band, (b) applying a notch filter at 50 Hz, (c) downsampling to 200 Hz, (d) applying third-order spatial gradient noise correction, and (e) removing cardiac and eye movement artifacts using the Signal Space Projection (Uusitalo & Ilmoniemi, 1997) routine available in Brainstorm. A 60-s segment was extracted for each subject, continuous or concatenated (minimum length of the continuous segment: 10 s), where no artifact was visibly present, ensuring with an EEG expert that the subject was awake during this segment. Following data preprocessing and sleep scoring, a total of 45 participants were ultimately included in the analysis (age: 29 ± 4 years, range: 20–38 years, M: 10). Notably, one participant was excluded due to sleeping during the acquisition, while 11 were excluded for coregistration, segmentation issues, or exceptionally noisy data.

#### Source space and forward model estimation.

For each participant, a T1-weighted, 3D turbo field-echo anatomical MRI was performed with a 3 T Ingenia CX Philips scanner (Philips Medical Systems, Best, The Netherlands). FreeSurfer (Dale et al., 1999) was used for subsequent brain segmentation and reconstruction of the cortical surfaces. The coregistration of MEG sensors with anatomical MRI and analysis for creating the source model and forward model were performed in Brainstorm (Tadel et al., 2011). The cortical mesh of the middle layer (white/gray matter interface), equidistant between the white matter and pial surfaces and comprising approximately 300,000 vertices, was considered as the source space. Additionally, the two hippocampi from the subcortical structures were included, each hippocampus consisting of around 3,000–4,000 vertices depending on the subject’s anatomy. For the cortex and hippocampus, sources were placed on the surface of the structures with a fixed orientation orthogonal to the surface at each point. The cortical and hippocampal surfaces were then combined as the source space and was downsampled to approximately 8,000 vertices. The forward model was computed using OpenMEEG software (Gramfort et al., 2010; Kybic et al., 2005) implemented in Brainstorm. We used a three-layer Boundary Element model (BEM) consisting of brain, skull, and scalp surfaces with conductivity values of 0.33, 0.0165, and 0.33 S m^−1^ (Zhang et al., 2006).

#### MEG source imaging using wMEM.

The MEG inverse problem was solved using the maximum entropy on the mean (MEM) framework (Amblard et al., 2004), a Bayesian approach validated in the context of EEG/MEG source imaging (Chowdhury et al., 2013). The key feature of MEM is a spatial prior model, assuming that brain activity is organized within cortical parcels, where the activity of every parcel is tuned by the probability of activation of a hidden state variable. When the parcel is active, a Gaussian prior is assumed to model a priori the activity within the parcel. Starting from such a prior “reference” distribution, inference is then obtained by maximizing the relative entropy to the prior. MEM can either switch off or switch on the corresponding parcels during the localization process while allowing local contrast along the cortical surface within the active parcels. wMEM is a variant of the MEM method specifically designed to localize brain oscillatory patterns (Afnan et al., 2023; Amblard et al., 2004; Lina et al., 2014). wMEM applies a discrete wavelet transformation (Daubechies wavelets) to characterize the oscillatory patterns in the data before applying the MEM solver for source imaging (Lina et al., 2014). wMEM was validated for localizing oscillatory patterns at seizure onset (Pellegrino et al., 2016), interictal bursts of high-frequency oscillations (Avigdor et al., 2021; von Ellenrieder et al., 2016), and MEG resting-state fluctuations (Aydin et al., 2020). We proposed and implemented several changes in standard wMEM to localize specifically oscillatory patterns in the resting state (details in Afnan et al., 2023), and evaluated the accuracy of reconstructions with the MNI iEEG atlas. In the current study, we used the wMEM version proposed by Afnan et al. (2023), adding the depth weighting parameter proposed and validated in Afnan et al. (2024) to localize deep brain activity more accurately.

To estimate a noise-covariance model from resting-state data, we created a quasisynthetic baseline from the signal of interest to compute the noise covariance by randomly shuffling the Fourier phase at each frequency (Prichard & Theiler, 1994). We employed a sliding window approach (window length: 1 s) to generate the baseline, ensuring a more precise estimation of the noise covariance matrix for each wavelet sample across the time scales (Afnan et al., 2023). wMEM implementation is available within the *BEst* plugin of Brainstorm software (https://neuroimage.usc.edu/brainstorm/Tutorials/TutBEst/).

#### Estimation of virtual iEEG data from the MEG source map.

MEG measures current densities (in nanoampere-meters) after source imaging, while iEEG records electrical potentials in microvolts. For a quantitative comparison, we converted MEG-reconstructed source maps into iEEG channel space by estimating corresponding electrical potentials for each electrode channel on the iEEG atlas (Abdallah et al., 2022; Grova et al., 2016). This involved localizing iEEG channels in the native MRI of MEG healthy subjects, by co-registering each subject MRI with the ICBM152 template using Minctracc (Collins et al., 1994), and applying a linear and nonlinear transformation to align electrode coordinates from the iEEG atlas to each subject’s anatomy. More details about this projection can be found in the Supporting Information S1. For each source map acquired from all 45 participants, we obtained MEG data converted into microvolts to the corresponding locations of 1,712 channels available in the iEEG atlas. This resulted in a larger number of MEG channels compared with the iEEG atlas (1,712 channels in the iEEG atlas vs. 1,712 × 45 channels in MEG). We used a bipolar montage for both iEEG and MEG-converted virtual iEEG. In our comparison of MEG-estimated oscillations with the iEEG atlas in Afnan et al. (2023), we used a common average montage and found similar results for a bipolar montage. However, we used a bipolar montage for the connectivity analysis as a common average montage can introduce spurious connections between channels (Bastos & Schoffelen, 2016; Shi et al., 2024).

#### Construction of reliable MEG connectomes using bootstrap resampling.

**Figure 1.**
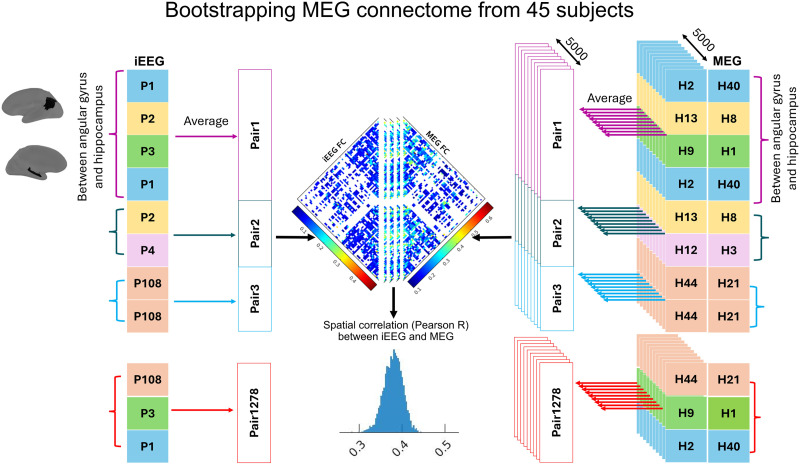
The iEEG connectome consists of connectivity metrics between pairs of channels, obtained from a total of 110 patients. In each of 45 MEG connectomes, all connections originated from a single subject. To generate a new MEG connectome comparable with the original iEEG connectome, MEG subjects were randomly chosen to contribute connections between ROIs while preserving spatial information. This process was repeated for all ROI pairs, resulting in a bootstrap-resampled MEG connectome, mimicking the same subjects’ group distribution as our original iEEG connectome. The spatial Pearson correlation between the original iEEG connectome and the bootstrap-resampled MEG connectome was computed. This overall process was iterated 5,000 times, yielding 5,000 correlation values.

We constructed 45 MEG connectomes, retaining only the connections present in the iEEG connectome (see Supporting Information Figure S1). The key contrast between the iEEG connectome and MEG connectomes is that the iEEG connectome can reflect contributions from multiple subjects, while each MEG connectome represents contributions solely from one subject. To address this, we employed a bootstrapping approach to develop an MEG connectome contributed by a group of participants, similar to the one considered when using the iEEG atlas. The iEEG connectome consists of connectivity metrics between channel pairs, obtained by pooling the contribution from 110 patients. For example, consider two ROIs—the hippocampus and angular gyrus, including four iEEG pairs of electrodes: The first and fourth connection pairs were obtained from one subject (iEEG_subject-1), while the second was obtained from iEEG_subject-2 and the third one from iEEG_subject-3. On the other hand, in each of 45 MEG connectomes, all connections would originate from a single subject. To create an MEG connectome mimicking the one obtained when using the iEEG atlas, we randomly select MEG subjects to contribute connection pairs between these two ROIs (hippocampus and angular gyrus). For instance, the first and fourth connection pairs came from one randomly selected MEG subject (e.g., MEG_subject-40), while the second and third connections were sampled from two other randomly selected MEG subjects (e.g., MEG_subject-8 and MEG_subject-1). As illustrated in Figure 1, this process was repeated for all ROI pairs to generate a bootstrap resampled MEG connectome, mimicking the same subject group distribution as our original iEEG connectome. This overall process was repeated 5,000 times and resulted in 5,000 bootstrap resampled MEG connectomes (Figure 1).

### Cross-Modal Correlation

We computed the spatial Pearson correlation between the original iEEG connectome and the 5,000 bootstrap resampled MEG connectomes. As a result, we obtained 5,000 Pearson correlation values, representing the spatial cross-modal correlation between iEEG and MEG data. To statistically assess the significance of cross-modal correlations, we also generated 5,000 cross-modal correlation values to build an empirical null distribution. To do so, for each iteration, we permuted randomly the anatomical labels of the channel pairs in the bootstrapped MEG connectomes, therefore effectively destroying the underlying spatial correlation structure. Then, we calculated the Pearson correlation between the iEEG connectome and the spatially permuted MEG connectomes, creating an empirical null distribution from those 5,000 correlation values. We defined a range for the null distribution, known as the region of practical equivalence, which included 95% of the distribution centered around the median of the null. A cross-modal (MEG connectome-iEEG connectome) correlation was considered significant if less than 2.5% of the actual distribution lay inside the null range (equivalent to a 5% two-tailed threshold, with 2.5% in each tail).

### Estimation of Connectivity Metrics

For analyzing electrophysiological data, various connectivity metrics are available, mainly classified into two categories: amplitude-based and phase-based metrics. In this study, we employed a widely used amplitude-based metric—the *AEC* (Brookes et al., 2011b) and a phase-based metric, the *PLV* (Mormann et al., 2000). Additionally, we utilized two metrics that correct/remove zero-lag connectivity: *OAEC* (Hipp et al., 2012) and a modified version of the *wPLI* (Vinck et al., 2011), which was modified to consider only the phase information.

Let us consider two signals *X* and *Y*. To obtain their corresponding amplitude envelope and instantaneous phases, we computed the Hilbert transform for the entire 60-s signals. The Hilbert transform was initially calculated for each 0.5-Hz frequency band and then averaged to obtain one transform for six canonical frequency bands (delta [0.5–4 Hz], theta [4–8 Hz], alpha [8–13 Hz], beta [13–30 Hz], low gamma [30–55 Hz], and high gamma [55–80 Hz]; Aydin et al., 2020).

*X*_*BP,H*_ and *Y*_*BP,H*_ (*BP* stands for bandpass and *H* stands for Hilbert) are the Hilbert analytical signals of each narrow frequency band for signals *X* and *Y*, described as *X*_*BP,H*_(*t*) = |*X*_*BP,H*_(*t*)|*e*^*jφ_X_*(*t*)^ = *A*_*X*_(*t*)*e*^*jφ_X_*(*t*)^ and *Y*_*BP,H*_(*t*) = |*Y*_*BP,H*_(*t*)|*e*^*jφ_Y_*(*t*)^ = *A*_*Y*_(*t*)*e*^*jφ_Y_*(*t*)^, respectively. Here, *A*_*X*_(*t*) and *A*_*Y*_(*t*) denote the instantaneous amplitude of *X*_*BP,H*_(*t*) and *Y*_*BP,H*_(*t*), *φ_X_*(*t*) and *φ_Y_*(*t*) denote the instantaneous phase of *X*_*BP,H*_(*t*) and *Y*_*BP,H*_(*t*), respectively. We considered the whole 60-s dataset to estimate AEC and OAEC. For PLV and wPLI, we used 6-s epochs and averaged the connectivity over the epochs.

#### AEC.

AEC between two signals, *X* and *Y*, is obtained by computing the Pearson correlation between the envelopes of *X*_*BP,H*_ and *Y*_*BP,H*_. (Hipp et al., 2012).

$$AEC=\frac{\sum_{t=1}^{T}\left(A_{X}\left(t\right)-{\bar{A}}_{X}\right)\left(A_{Y}\left(t\right)-{\bar{A}}_{Y}\right)}{\sqrt{\sum_{t=1}^{T}{\left(A_{X}\left(t\right)-{\bar{A}}_{X}\right)}^{2}\sum_{t=1}^{T}{\left(A_{Y}\left(t\right)-{\bar{A}}_{Y}\right)}^{2}}},$$ (1)

where *T* is the length of the signal (we considered 60-s at 200-Hz sampling, *T* = 12,000 samples) and ${\bar{A}}_{X}$and ${\bar{A}}_{Y}$ are the mean values of *A*_*X*_(*t*) and *A*_*Y*_(*t*), respectively.

#### OAEC.

OAEC was proposed by Hipp et al. (2012) following a pairwise orthogonalization between two signals.

$$Y_{⟂X}=\text{imag}\left(Y_{BP,H}\frac{{X_{BP,H}}^{*}}{\left|X_{BP,H}\right|}\right)$$ (2)

$$X_{⟂Y}=\text{imag}\left(X_{BP,H}\frac{{Y_{BP,H}}^{*}}{\left|Y_{BP,H}\right|}\right)$$ (3)

Here, * means complex conjugate and *imag* means the imaginary part of the complex number.

We calculated the Pearson correlation between the envelopes of *X*_*BP,H*_ and *Y*_⟂*X*_. Similarly, the correlation between the envelopes of *Y*_*BP,H*_ and *X*_⟂*Y*_ is calculated and then the average of these two correlation values is considered as the final OAEC value.

#### PLV.

PLV was originally proposed in Lachaux et al. (1999) in the context of evoked activity considering a stable phase difference along trials. We calculated PLV for each epoch of 6 s using an extended definition of PLV (Equation 3), a version proposed by Mormann et al. (2000) in the context of resting-state data, by assessing phase locking as a stable phase difference over time:

$${PLV}_{X,Y}=\frac{1}{T}\left|\sum_{t=1}^{T}\exp\left(j\left(\varphi_{X}\left(t\right)-\varphi_{Y}\left(t\right)\right)\right)\right|,$$ (4)

where *T* is the length of the signal (we considered 6-s epochs at 200-Hz sampling, *T* = 1,200 samples); *j* denotes the imaginary unit; and *φ_X_*(*t*) and *φ_Y_*(*t*) are, respectively, the corresponding instantaneous phases of signals *X* and *Y* at time point *t*. We implemented PLV following the derivation proposed by Bruña et al. (2018) as:

$${PLV}_{X,Y}=\frac{1}{T}\left|\sum_{t=1}^{T}{\dot{X}}_{BP,H}\left(t\right).{\left({\dot{Y}}_{BP,H}\left(t\right)\right)}^{*}\right|,$$ (5)

where ${\dot{X}}_{BP,H}\left(t\right)=\frac{X_{BP,H}\left(t\right)}{\left|X_{BP,H}\left(t\right)\right|}$ and ${\dot{Y}}_{BP,H}\left(t\right)=\frac{Y_{BP,H}\left(t\right)}{\left|Y_{BP,H}\left(t\right)\right|}$.

Finally, we averaged the PLV values across all the epochs.

#### Modified Weighted Phase Locking Index (wPLI*).

The original formula of wPLI is proposed by Vinck et al. (2011):

$$\text{wPLI}=\frac{\left|\sum_{t=1}^{T}A_{X}\left(t\right)A_{Y}\left(t\right)\sin\left(\varphi_{X}\left(t\right)-\varphi_{Y}\left(t\right)\right)\right|}{\sum_{t=1}^{T}|A_{X}\left(t\right)A_{Y}\left(t\right)\sin\left(\varphi_{X}\left(t\right)-\varphi_{Y}\left(t\right)\right)|}$$ (6)

If we consider that the amplitudes of the channels in a particular frequency band are stable in time and not involved in the coupling of the channels, the coupling will be strictly described by the phase synchronization and driven by the constant phase shift. Under this assumption, the modified version of wPLI becomes:

$${\text{wPLI}}^{*}=\frac{\left|\sum_{t=1}^{T}\sin\left(\varphi_{X}\left(t\right)-\varphi_{Y}\left(t\right)\right)\right|}{\sum_{t=1}^{T}|\sin\left(\varphi_{X}\left(t\right)-\varphi_{Y}\left(t\right)\right)|},$$ (7)

where *T* is the length of the signal (we considered 6-s epochs at 200-Hz sampling, *T* = 1,200 samples). We averaged the wPLI* values across all the epochs.

The results obtained when considering the original wPLI formula are also provided in the Supporting Information.

## RESULTS

### Connectivity Estimated by MEG Versus iEEG

**Figure 2.**
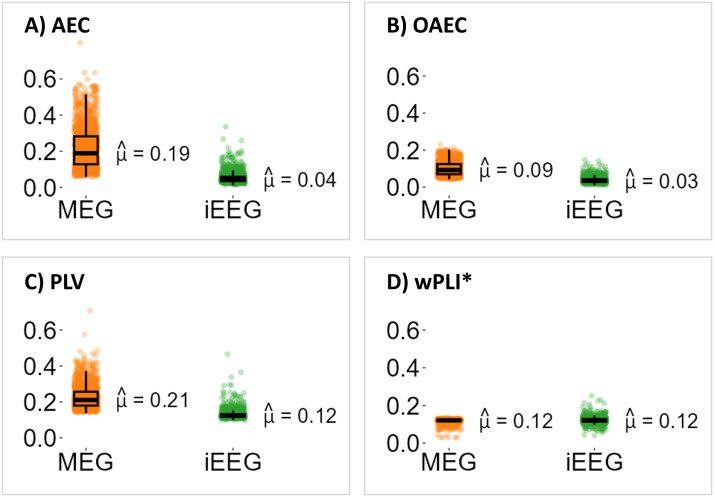
Connectivity averaged across frequency bands estimated by MEG and iEEG calculated using (A) *AEC*, (B) *OAEC*, (C) *PLV*, and (D) wPLI*. For iEEG, we considered all 1,278 iEEG ROI pairs available from all patients of the iEEG atlas and showed the averaged connectivity across six frequency bands. For MEG, we considered 1,278 virtual iEEG ROI pairs from each of the 45 subjects and showed the averaged connectivity across 45 subjects and six frequency bands. The median value of each distribution is displayed.

Figure 2 shows the distribution of connectivity values estimated by MEG and iEEG for four connectivity metrics over the whole available iEEG connectome. Here, we used all the original MEG connectomes estimated from 45 subjects, not the resampled MEG connectome as described in Construction of reliable MEG connectomes using bootstrap resampling. In this figure, iEEG connectivities were averaged across six frequency bands and MEG connectivities were averaged across 45 subjects and six frequency bands. Each boxplot shows 1,278 connectivity values (from 1,278 ROI pairs) for iEEG and MEG.

This figure provides a general overview of the scale of connectivity values one could expect from MEG versus iEEG connectomes. We found large differences in *AEC* and *PLV* connectivity values estimated from MEG when compared with iEEG, given that those two metrics are sensitive to volume conduction leakage. For *OAEC* and wPLI*, which removed zero-lag connectivity, MEG and iEEG connectivity values were found within a more similar range, but both were very low. We will investigate those connectivity values as a function of the distance between two ROIs in Connectivity as a Function of Distance Between Two ROIs.

### Cross-Modal Spatial Correlation: AEC and OAEC

**Figure 3.**
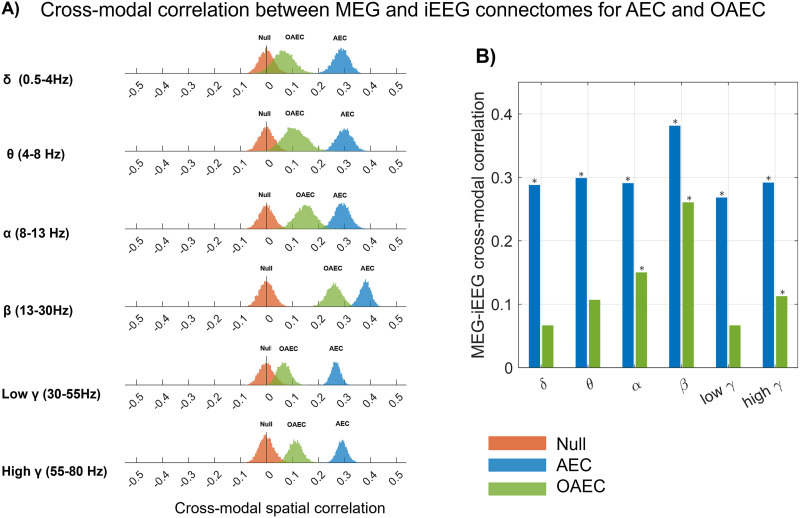
(A) Distribution of cross-modal correlations as well as the null distribution (red) between MEG and iEEG for six frequency bands calculated for AEC (blue) and OAEC (green). (B) The medians of the distribution of cross-modal correlations are shown in the bar plot. The correlation was considered significant if its overlap with the null range was less than 2.5% (equivalent to a 5% two-tailed threshold, with 2.5% in each tail). The frequency bands that showed significantly higher correlations than the null distribution are marked with an asterisk (*).

After considering 5,000 bootstrap resampled MEG connectomes mimicking the same spatial/population distribution as our original iEEG connectome, Figure 3A presents the results of cross-modal spatial correlations between MEG and iEEG connectomes for six frequency bands calculated from *AEC* and *OAEC*, when compared with null distributions obtained by spatial permutation of the ROI pairs. The differences between the cross-modal correlations and the null distributions were as follows: For AEC: *δ*: 0.29 ± 0.03, *θ*: 0.30 ± 0.03, *α*: 0.29 ± 0.03, *β*: 0.38 ± 0.02, low *γ*: 0.27 ± 0.02, and high *γ*: 0.29 ± 0.02 (values reported as median ± median absolute deviation). For OAEC: *δ*: 0.06 ± 0.03, *θ*: 0.11 ± 0.03, *α*: 0.15 ± 0.03, *β*: 0.26 ± 0.03, low *γ*: 0.07 ± 0.03, and high *γ*: 0.11 ± 0.03.

Figure 3B displays the median value of the distribution for each cross-modal correlation and highlights correlations that were significantly larger than null. Unless specified otherwise, the correlation was considered significant if its overlap with the null range was less than 2.5% (equivalent to a 5% two-tailed threshold, with 2.5% in each tail). When considering the *AEC* metric, MEG-estimated connectomes were moderately correlated with iEEG connectomes across all frequency bands (∼0.25–0.37), with the highest correlation observed in the beta band (0.37). When considering the *OAEC* metric, the median of the cross-modal correlations decreased compared with *AEC*, but they remained significantly higher than the null distribution in the alpha, beta, and high gamma bands, with the highest correlation still observed in the beta band (0.26).

**Figure 4.**
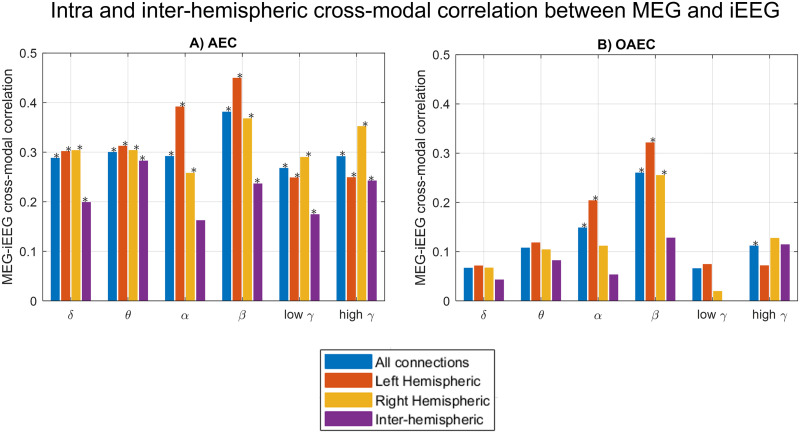
The median of the distribution of cross-modal correlations is depicted, considering all connections, intrahemispheric connections, and interhemispheric connections for (A) AEC and (B) OAEC. The correlation was considered significant if its overlap with the null range was less than 2.5% (equivalent to a 5% two-tailed threshold, with 2.5% in each tail). Frequency bands with significantly higher correlations than the null distribution are marked with an asterisk (*).

Figure 4 further presents intrahemispheric and interhemispheric connections, alongside all available connections. Similarly to Figure 3, intrahemispheric connectomes estimated from MEG using *AEC* were moderately correlated to those from iEEG across all frequency bands (significantly higher than the null distribution). Interhemispheric cross-modal correlations were significantly higher than the null distribution in all bands except alpha. For *OAEC*, inter- and intrahemispheric correlations decreased compared with *AEC* in all frequency bands (Figure 4B). Intrahemispheric correlations were significantly higher than the null distribution for the beta band, whereas interhemispheric correlations were not found statistically significant in any band.

To assess the reliability of our findings, we conducted a supplementary investigation using a bootstrap resampling strategy to generate the iEEG connectomes. We divided the 60 s of iEEG data into ten 6-s segments and performed bootstrapping with replacement 100 times (similarly to the approach we proposed in Aydin et al., 2020). More details are provided in Supporting Information S2. The spatial correlations between MEG and iEEG for AEC and OAEC, computed from the 100 bootstrapped iEEG datasets, are shown in Supporting Information Figure S2. The patterns for AEC and OAEC were similar to those shown in Figure 3B, suggesting robustness of our findings.

### Cross-Modal Spatial Correlation: *PLV* and wPLI*

**Figure 5.**
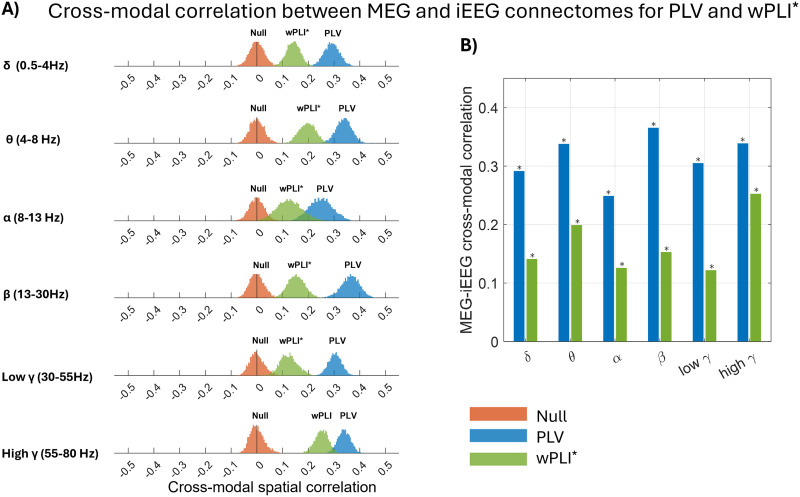
Distribution of cross-modal correlations and the null distribution (red) between MEG and iEEG for six frequency bands calculated for *PLV* (blue) and wPLI* (green). (A) The medians of the distribution of cross-modal correlations were shown as a bar plot. (B) The correlation was considered significant if its overlap with the null range was less than 2.5% (equivalent to a 5% two-tailed threshold, with 2.5% in each tail). The frequency bands that showed significantly higher correlations than the null distribution were marked with an asterisk (*).

Figure 5A presents the cross-modal correlation between MEG and iEEG connectomes, depicted for six frequency bands, using the connectivity metrics *PLV* and wPLI*. *PLV* exhibited moderate cross-modal spatial correlation across all frequency bands, with the highest correlation in the beta band. The differences between the cross-modal correlation and the null distribution for all frequency bands were as follows: *δ*: 0.29 ± 0.03, *θ*: 0.34 ± 0.03, *α*: 0.25 ± 0.04, *β*: 0.36 ± 0.03, low *γ*: 0.31 ± 0.03, and high *γ*: 0.34 ± 0.03. For wPLI*, these differences were: *δ*: 0.14 ± 0.03, *θ*: 0.2 ± 0.03, *α*: 0.13 ± 0.04, *β*: 0.15 ± 0.03, low *γ*: 0.12 ± 0.03, and high *γ*: 0.25 ± 0.03.

In Figure 5B, the median values of each cross-modal correlation distribution are presented as a bar plot for both *PLV* and wPLI*, highlighting correlations significantly larger than the empirical null distribution. For *PLV*, MEG-estimated connectomes were moderately correlated to iEEG connectomes across all frequency bands (∼0.3). With wPLI*, the median of cross-modal correlations decreased compared with *PLV*, but remained significantly higher than the null distribution for all frequency bands.

The spatial correlations between MEG and iEEG for intra- and interhemispheric connectomes for *PLV* and wPLI* are presented in Supporting Information Figure S3. For *PLV*, significant cross-modal correlations were observed for both intrahemispheric connectomes in all frequency bands. Interhemispheric correlations were statistically significant in all frequency bands except alpha. Interestingly for wPLI*, the interhemispheric correlations were statistically significantly higher than the null distribution in all frequency bands, whereas the left hemispheric correlations were not significantly higher than null in any band. Moreover, the right hemispheric correlations were found higher than the left hemispheric correlations in all frequency bands. To further investigate this surprising result, we also evaluated the raw wPLI* separately for iEEG and MEG for left-hemispheric versus right-hemispheric connectomes, and the distributions of wPLI* did not show such laterality differences.

To assess if the left versus right and interhemispheric asymmetry found by wPLI* could be influenced by the choice of the source imaging method (wMEM), we also repeated this analysis using another standard source imaging method, the minimum norm estimate (Hämäläinen & Ilmoniemi, 1994). We found a similar trend, that is, the interhemispheric correlations were higher than the left/right hemispheric correlations and the right hemispheric correlations were higher than the left hemispheric correlations (result not shown). In Supporting Information Figure S4, we also provided the correlations between MEG and iEEG for intra- and interhemispheric connectomes for *wPLI*, calculated using the original definition, which also included envelope amplitudes, as described in Equation 7. Using this implementation, the resulting correlations were overall very low and not statistically significant in any frequency bands.

### Cross-Modal Spatial Correlation for Superficial Versus Deep Sources

**Figure 6.**
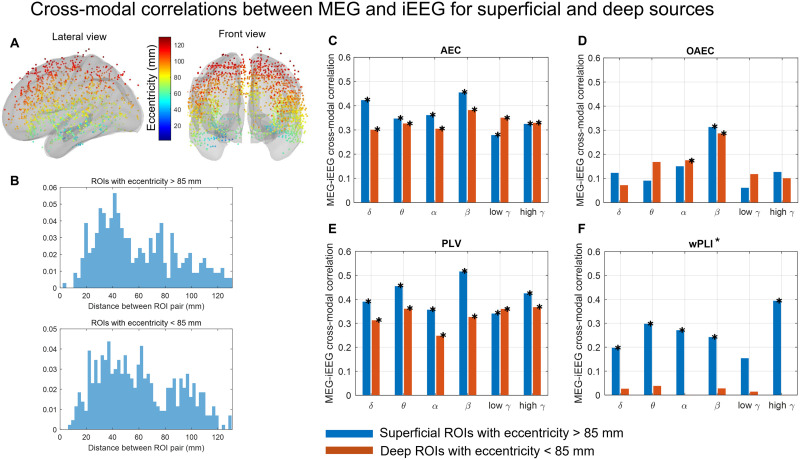
(A) Eccentricity of iEEG channels shown on the brain cortex with 80% transparency to ensure all deep iEEG channels are visible. (B) Distribution of the distances between ROI pairs for all pairs exhibiting either an eccentricity > 85 mm (top) or < 85 mm (bottom). The cross-modal correlation between MEG and iEEG for two groups (both eccentricity values > 85 mm in blue and both eccentricity values < 85 mm in red) for *AEC* (C), *OAEC* (D), *PLV* (E), and wPLI* (F). The correlation was considered significant if its overlap with the null range was less than 2.5% (equivalent to a 5% two-tailed threshold, with 2.5% in each tail). The frequency bands that showed significantly higher correlations than the null distribution were shown with an *.

In Figure 6, we investigated the cross-modal correlations between MEG and iEEG for superficial and deep ROI pairs. For each iEEG channel, we measured the eccentricity, defined as the distance between the channel location and the center of the head. Deep channels have therefore low eccentricity, and superficial channels have high eccentricity. Figure 6A shows the distribution of eccentricity values for all the channels, using 80% transparency of the cortical mesh so that all channels are visible in the figure. A threshold of 85-mm eccentricity was selected to classify the channels into superficial and deep channels. We used this threshold of 85 mm to have a similar number of ROI pairs in superficial versus deep connectomes. The distributions of the distance between ROI pairs for two groups (eccentricity > 85 mm for both ROIs of the pair and eccentricity < 85 mm for both ROIs of the pair) are shown in Figure 6B. The cross-modal correlations between MEG and iEEG connectomes for these two groups are depicted for the six frequency bands for *AEC, OAEC, PLV*, and wPLI* (Figure 6C–F). For *AEC* and *PLV*, the cross-modal correlations were significantly higher than the null distribution for all frequency bands for both groups. However, the cross-modal correlations for deep ROI pairs had a trend of decrease when compared with superficial ROI pairs for delta, theta, alpha, and beta bands. For *OAEC*, the cross-modal correlations were significantly higher than the null distribution in the beta band for both superficial and deep ROI pairs. On the other hand, the correlation for deep ROI pairs in the alpha band was found significantly higher than null, whereas the correlation for superficial ROI pairs did not reach the significant threshold. Interestingly for wPLI*, the correlations for superficial ROI pairs were significantly higher than the null distribution for all frequency bands except low gamma, whereas the correlations for deep ROI pairs were very low and did not reach the significant threshold in any frequency band.

### Connectivity as a Function of Distance Between Two ROIs

**Figure 7.**
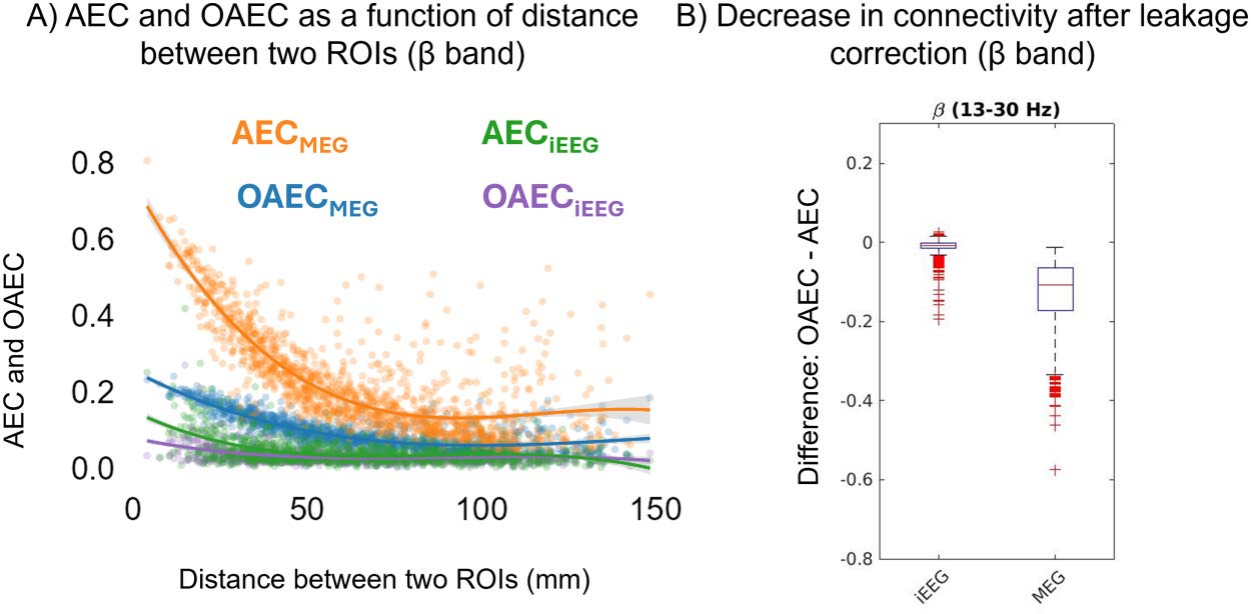
(A) *AEC* and *OAEC* as a function of distance between two ROIs plotted for iEEG and MEG in the beta band. (B) The distribution of differences between *OAEC* and *AEC* (*OAEC* minus *AEC*) for MEG and iEEG.

In this section, we investigated raw connectivity values from iEEG and MEG. Figure 7A shows the *AEC* and *OAEC* values as a function of the distance between two ROIs for iEEG and MEG for beta band results. For MEG, the connectivity values were averaged over 45 subjects. As expected, both *AEC* and *OAEC* decreased as a function of distance between the two ROIs. However, for *AEC*, MEG connectivity values were greater than iEEG. After orthogonalization, both MEG and iEEG connectivity values decreased, but the decrease in MEG was higher than iEEG, which was quantified and plotted in Figure 7B. The *AEC* and *OAEC* for all frequency bands are presented in Supporting Information Figure S5. Across all frequency bands, the reduction in MEG connectivity following orthogonalization exceeded that of iEEG (see Supporting Information Figure S6). The raw *PLV* and wPLI* values as a function of distance between ROIs for all frequency bands are presented in Supporting Information Figure S7. Similar to *AEC* and *OAEC, PLV* also decreased as a function of the distance between two ROIs for both MEG and iEEG. *PLV* estimated by MEG were greater than iEEG. However for wPLI*, the relationship of the values as a function of the distance between ROI pairs is not as clear as found for other metrics.

### Cross-Modal Spatial Correlation and the Number of Subjects Averaged in ROI Pairs

In previous results, we actually estimated the iEEG connectomes (real or virtual) by averaging all possible pairs of channels between each ROI pair (Construction of iEEG connectome). The results shown so far were produced using this criterion: at least one pair of channels connecting the ROI pair, which resulted in a connectome containing 1,278 ROI pairs (out of 2,888 possible ROI pairs, resulting in 44% coverage of the whole connectome). To assess the effect of the number of subjects having an ROI pair, we further investigated the cross-modal spatial correlations between MEG and iEEG connectomes while increasing the minimum number of subjects to be averaged for each ROI pair. However, increasing the minimum number of subjects in each ROI pair limits the coverage of the iEEG connectome we could consider (some ROI pairs have only one subject, some have two, etc.).

**Figure 8.**
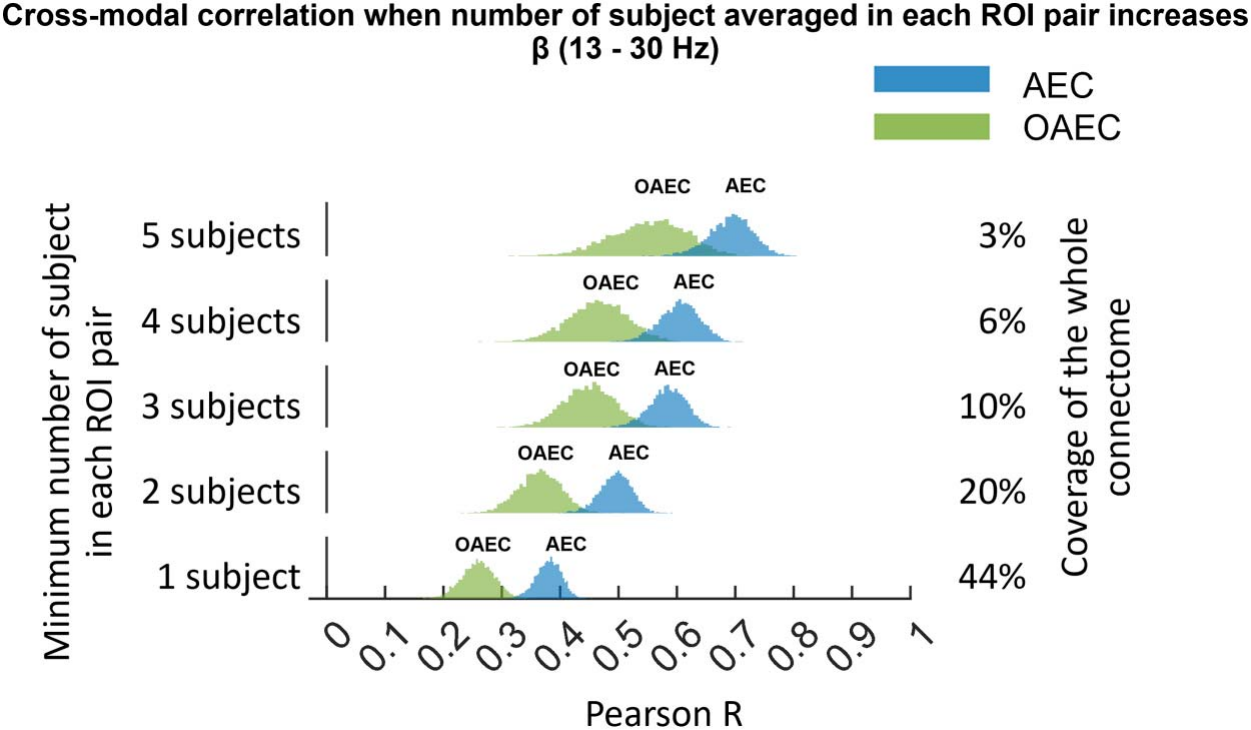
Distribution of cross-modal spatial correlations between MEG and iEEG connectomes in the beta band obtained using *AEC* and *OAEC* (obtained from 5,000 bootstrap MEG samples), as we increase the minimum number of subjects from one to five in each ROI pair. Increasing the minimum number of subjects in each ROI pair (as shown on the left) decreases the available coverage of the iEEG connectome from 44% to 3% (as shown on the right). For example, the bottom row displays histograms of the correlations between MEG and iEEG connectomes when the iEEG connectome was created with ROI pairs that include at least one patient, covering 44% of the connectome.

Figure 8 shows the cross-modal correlations for *AEC* and *OAEC* in the beta band as an example. On the *y*-axis, we show the minimum number of subjects on the left and the percentage coverage of the whole connectome on the right. The cross-modal correlations between MEG and iEEG increased as the minimum number of subjects included in each ROI pair increased. The lowest value of the minimum number of patients ( = 1) means including all possible ROI pairs, thus maximizing the connectome coverage. Using a minimum number of patients of one provided 1,278 ROI pairs, covering 44% of the whole connectome. Increasing the minimum number of patients in each ROI pair to two, three, four, and five decreased the connectome coverage to 20%, 10%, 6%, and 3% of the whole connectome, respectively. We did not show results for a minimum number of patients greater than five, as the coverage of the connectome decreases to less than 1%.

The cross-modal correlations for six frequency bands as we increased the minimum number of subjects in each ROI pair are shown in Supporting Information Figure S8. A similar trend of increased cross-modal correlation was found for *AEC* and *OAEC* in delta, theta, alpha, beta, and low gamma bands.

In Supporting Information Figure S9, the cross-modal correlations obtained using *PLV* and wPLI* are shown for all six frequency bands as we increased the minimum number of subjects in each ROI pair. For all frequency bands, the cross-modal correlations for *PLV* increased as the minimum number of subjects in each ROI pair increased. However, for wPLI*, we did not find the trend of increasing cross-modal correlation as we increased the minimum number of subjects.

## DISCUSSION

Our objective was to validate the ability of MEG to estimate resting-state connectomes for healthy subjects by comparing them with an iEEG atlas. To compare the two modalities in the same space, we converted MEG sources into virtual iEEG potentials (Abdallah et al., 2022; Grova et al., 2016). As opposed to estimating virtual channels using beamforming approaches (Tamilia et al., 2021), our strategy is to combine an MEG source imaging method that was evaluated for its ability to localize accurately resting-state MEG data and notably oscillations, the wMEM (Afnan et al., 2023), followed by applying an iEEG forward problem to estimate virtual iEEG potentials in microvolts that correspond to our MEG sources (Abdallah et al., 2022; Grova et al., 2016). This offers a solid quantification approach to compare MEG sources (estimated by solving an inverse problem) with actual iEEG in situ recordings. Consequently, the two modalities were associated with different distributions of available data when estimating connectomes. For MEG, we were able to estimate 45 connectomes, each coming from one subject and providing virtual iEEG data on all channels of the iEEG atlas. Thus, each connectome was contributed by the same subject. In contrast, when considering the multicentric iEEG atlas, we were able to estimate one iEEG connectome, which was obtained by pooling data from 110 subjects, each subject contributing to a subset of the connectome. To address the discrepancy between MEG and iEEG data distribution when estimating connectomes, we proposed a bootstrap resampling approach to create an MEG connectome spatially sampled in the same way as the iEEG connectome, such that each bootstrapped MEG connectome was built by pooling data in a similar way of constructing iEEG connectome.

Spatial cross-modal correlations between MEG and iEEG ranging from ∼0.25 to 0.38 were observed for *AEC* and *PLV*. As expected, we found that considering *OAEC* or wPLI*, as metrics that correct/remove zero-lag connectivity, led to a reduction in cross-modal correlations. This highlights the trade-off: While MEG may exhibit more connectivity due to source leakage, removing zero-lag connectivity also eliminates genuine connections, thereby decreasing overall cross-modal correlation. These results are also supported by the fact that even for the original iEEG, we found a small decrease in connectivity when removing zero-lag connectivity. This suggests that the observed connectivity is more likely to be genuine time-locked zero phase connectivity (see Figure 7), as iEEG, being local in situ measurements, are less sensitive to source leakage and volume conduction (O’Reilly & Elsabbagh, 2021). These findings are consistent with prior studies (Colclough et al., 2016; Palva et al., 2018) that were conducted using simulations. In addition, there was a general trend of higher cross-modal spatial correlations between MEG and iEEG for superficial ROI pairs compared with deep ROI pairs, with a few exceptions. The decrease in correlations for deep ROI pairs was more prominent in phase-based metrics compared with amplitude-based metrics. The differences in cross-modal correlations for amplitude- and phase-based metrics also highlight that those metrics are capturing distinct information, more likely supported by different underlying mechanisms (Siems & Siegel, 2020).

Moreover, to the best of our knowledge, our study is the first to quantify the extent of overestimation of MEG connectivity when compared with ground truth iEEG data, at the population level. This overestimation was consistently observed across all frequency bands. This is also the first study to quantify and compare MEG and iEEG connectomes. For both MEG and iEEG, *AEC* and *PLV* values decreased with increasing distance between two ROIs, consistent with previous studies with animal electrophysiology (Leopold et al., 2003) and human iEEG (Arnulfo et al., 2015). The decrease in connectivity with increasing distance between regions was also found for *OAEC*, but not much for wPLI* for which we mainly found very small connectivity values on those resting-state data.

### Moderate Correlations Between MEG and iEEG Connectome

The cross-modal correlations between MEG and iEEG connectomes for different metrics across all frequency bands were generally moderate to low (∼0.12 to 0.37 for all connections, when statistically significant). It is however important to note that we did not expect very high correlations between these two modalities due to several factors contributing to the differences, such as: (a) nonsimultaneous data from two different groups; (b) different subjects contributing to the single iEEG connectome versus each of the 45 participants contributing to the MEG connectome (45 MEG connectomes), although we attempted to address this by adopting a bootstrap resampling approach; and (c) different levels of averaging for different ROI pairs (ranging between 1 and 217 channel pairs). Considering these variabilities, the cross-modal correlations found between MEG and iEEG suggest that we can recover some relevant connectivity patterns from MEG. However, since these correlations are moderate to low and vary across different metrics, the choice of metrics is important and the results of MEG connectivity should be interpreted with caution.

For connectivity metrics that do not remove zero-lag connectivity (*AEC*/*PLV*), moderate but significant cross-modal correlations (∼0.25 to 0.45) between MEG and iEEG were found in all frequency bands. For connectivity metrics that corrected/removed the zero-lag connectivity, the cross-modal correlations decreased. When compared with the empirical null distribution of cross-modal correlation, the resulting correlations were found significant in alpha and beta bands for *OAEC*. For wPLI*, although the correlations were low (∼0.15 to 0.25), they were statistically significant in all frequency bands. Overall, we observed the highest cross-modal correlation in the beta band for *AEC, OAEC*, and *PLV*.

Previous studies suggested that intrinsic networks estimated by MEG show the strongest correlation with fMRI-derived networks in the alpha and beta bands estimated by AEC (Brookes et al., 2011b). They proposed that the frequency of the amplitude envelope in these bands might better match slower fMRI signal fluctuations. Similarly, Wirsich et al. (2020) estimated the cross-modal correlation between simultaneous fMRI and EEG connectome generated using imaginary coherence (Nolte et al., 2004) and reported similar cross-modal correlations as those we obtained (∼0.29 to 0.36), with the highest correlations also found in the beta band. They found consistent results with multiple datasets using imaginary coherence (Wirsich et al., 2017, 2020) and across different MRI systems using *AEC*/*OAEC* and imaginary coherence (Wirsich et al., 2021). In Wirsich et al. (2021), the cross-modal correlations between fMRI and EEG connectivity using *OAEC* were lower compared with *AEC*, which is consistent with our findings.

In contrast to the studies mentioned above, where two modalities were compared across various frequency bands, Shafiei et al. (2022) adopted a different approach to compare fMRI and MEG-derived connectivity using OAEC. They illustrated that MEG oscillations across multiple bands might combine to give rise to the fMRI functional networks. They reported that while all frequency bands contribute to forming fMRI networks, beta band connectivity made the largest contribution, followed by theta and alpha connectivity. This was consistent with previous studies (Brookes et al., 2011b; Deligianni et al., 2014; Sadaghiani et al., 2022) suggesting that the frequency of the slower oscillation (i.e., extracted from the envelope of the alpha/beta oscillations) would be more similar to the fMRI fluctuations.

Unlike those studies, which compared hemodynamic correlations measured with fMRI with EEG/MEG connectivities, known to capture different brain mechanisms at varying time scales, we aimed to compare two modalities, iEEG and MEG, capturing essentially the same brain dynamics at the same time scale. For this reason, it is surprising that the cross-modal correlations between MEG and iEEG were in a similar range as, and not higher than those observed, in studies comparing fMRI with EEG/MEG. The reasons for the frequency-specific, cross-modal similarities, as well as why correlations in the beta band were higher than in other bands, remain unclear and pose important questions for future studies.

However, it is interesting to observe higher cross-modal correlations between MEG and iEEG for *AEC, OAEC*, and *PLV* when we increase the number of subjects to average in each ROI pair to construct the connectome, but at the cost of reducing overall connectome coverage. For instance, when we ensured at least three subjects in each ROI pair to construct the connectome, the cross-modal correlations between MEG and iEEG for *AEC, PLV*, and *OAEC* were 0.6, 0.6, and 0.45 (Figure 8, Supporting Information Figures S8 and S9), respectively, compared with 0.37, 0.36, and 0.26 (Figures 3 and 5), values found when we used at least one subject for each ROI pair. However, even if we found larger cross-modal correlations when averaging more subjects, we could hardly consider this a comprehensive connectome because it covered only 10% of the entire connectome. Thus, we chose to use at least one subject to create the connectome, which maximized connectome coverage (44%). More surprisingly, wPLI* did not follow this trend.

Increasing the minimum number of subjects to create the connectome likely removes noisy connections contributed by single subjects. In another study using simultaneous EEG-fMRI (Wirsich et al., 2021), the authors compared cross-modal correlations between EEG and fMRI. Although the data were simultaneous, the cross-modal correlation between EEG and fMRI for individual subjects was very low across all frequency bands. They found moderate cross-modal correlations (∼0.3 to 0.4) when averaging at least 7–12 subjects. This finding is interesting, and we expect that cross-modal correlations between MEG and iEEG connectomes could similarly benefit from averaging more subjects to reduce noisy connections. However, drawing such conclusions from our iEEG data is challenging, as increasing the number of subjects in each ROI pair drastically decreases the coverage of the iEEG connectome. We would require more subjects in the iEEG atlas to fully assess this. Nevertheless, our results suggest that cross-modal correlations between MEG and iEEG connectomes may increase when the iEEG connectome includes more subjects in each ROI pair.

### Compromise Between Removing Spurious Connectivity and Genuine Zero-Lag Connectivity

The issue of source leakage or volume conduction in EEG/MEG connectivity, as well as the search for the best connectivity metric, has been a topic of discussion for the past few years. Several studies reported the source leakage issue involved with EEG/MEG-derived connectivity and recommended to use connectivity metrics that remove zero-lag connections for obtaining interpretable results (Hipp et al., 2012; Palva & Palva, 2012; Schoffelen & Gross, 2009). Garcés et al. (2016) investigated the test–retest reliability of MEG resting-state functional connectivity for *PLV, PLI, AEC*, and *OAEC* by evaluating the within- and between-subject variability using the intraclass correlation coefficient. They found higher reliability for *PLV* across theta to gamma bands and for *OAEC* and *AEC* in the beta band. They suggested that volume conduction effects could contribute to high reliability for *PLV* and *AEC*. Rizkallah et al. (2020) compared resting-state EEG/MEG connectomes with fMRI-derived connectomes and reported significant correlations (but very low) between EEG/MEG connectomes and fMRI connectomes for *AEC* and *PLV*, whereas metrics that remove zero-lag connectivity exhibited no significant spatial cross-modal correlations. Finger et al. (2016) proposed a computational model and structural data from diffusion MRI tractography to simulate functional connectivity in the alpha band and compared it with empirical EEG functional connectivity for six connectivity metrics. They found high correlations between simulated and empirical functional connectivity for *PLV* and coherence (∼0.6), whereas the other metrics that remove zero-lag connectivity including *PLI* and *wPLI* exhibited low correlations (∼0.18). While they did not rule out the possibility that the high correlation found for *PLV* and coherence could be influenced by volume conduction, they questioned the use of metrics that remove zero-lag connectivity, as they might eliminate genuine neural synchrony mainly driven by the underlying anatomical structure.

Unlike previous studies that attempted to address this issue either through simulations or by comparing modalities known to detect different brain mechanisms at varying time scales, such as EEG/MEG with fMRI, we compared the MEG connectome with the iEEG connectome, both of which record similar brain activity. When compared with the iEEG connectome, we observed moderate correlations between the MEG connectome and iEEG connectome for *AEC* and *PLV*. The comparison of raw connectivity values revealed that MEG exhibited higher connectivity than iEEG across all frequency bands, confirming the inflated connectivity associated with EEG/MEG source leakage and volume conduction. However, for *OAEC* and wPLI*, metrics that remove or correct zero-lag connectivity (recommended to avoid spurious connectivity), although the raw connectivity values were found more similar for both modalities (Figure 2), the spatial correlations between MEG and iEEG connectomes decreased (Figures 3 and 5). In addition, the quantification of the difference between *AEC* and *OAEC* for MEG versus iEEG provided a clear representation that MEG indeed exhibits more zero-lag connections compared with iEEG, consistently observed across all frequency bands (Figure 7, Supporting Information Figure S6).

Thus the question of which metric is best for EEG/MEG connectivity analysis remains difficult to answer. The choice of metric should depend on the research question. Based on our findings, for the resting-state connectivity analysis at the connectome level, it may be important to use metrics that preserve zero-lag connections. If the study necessitates removing volume conduction, *OAEC* could be a good compromise as it corrects for zero-lag connectivity and also shows significant correlations between MEG and iEEG in the alpha and beta bands. Moreover, the cross-modal correlations for *OAEC* increased when we increased the minimum number of subjects to create the iEEG connectome, a trend also observed for *AEC* and *PLV*. For wPLI*, we found significantly higher cross-modal correlations in all frequency bands, which were consistently significant when only superficial ROIs were included. However, wPLI* for deep ROI pairs showed very low and statistically nonsignificant correlations in all frequency bands. The number of subjects averaged in each ROI pair also did not affect wPLI* results, unlike what was observed for *AEC, OAEC*, and *PLV* (Supporting Information Figures S8 and S9). Unlike *AEC, OAEC*, and *PLV*, the wPLI* metric was also not affected by the distance between the two ROIs (Supporting Information Figure S7). Furthermore, it remains unclear why there was asymmetry in cross-modal correlations computed for left, right, and interhemispheric connectomes using wPLI*. These surprising trends reported using wPLI* should be further investigated, and this metric should therefore be considered with caution. Carefully assessing the reliability of wPLI* but also other metrics, using test/retest reliability (Garcés et al., 2016) could be very important, but this was out of the scope of present study. It is important to note that we only considered the phase information to calculate the wPLI*. When analyzed with the original definition, which includes the amplitude information of Hilbert, the cross-modal correlations were very low (clearly lower than wPLI* results) and not significant in any frequency band, suggesting some instabilities when considered this family of metric.

### Cross-Modal Correlations for Deep Versus Superficial ROIs

The raw connectivity values for superficial versus deep ROIs had similar distributions for both iEEG and MEG. However, the correlations between MEG and iEEG connectomes for superficial ROIs were higher than for deep ROIs. This is not surprising because detecting and localizing deep subcortical sources by EEG/MEG is challenging for several reasons, such as the rapid attenuation of signals generated from deep structures with the distance of the generator from the EEG/MEG sensors, a phenomenon more pronounced for MEG when considering gradiometers (Barkley & Baumgartner, 2003; Malmivuo & Plonsey, 1995). The spatial configuration of the deep/subcortical structures also results in signal cancellation (Lorente De Nó, 1947; Murakami & Okada, 2006) and are difficult to detect by distant sensors. In Afnan et al. (2024), we proposed a depth-weighting parameter in MEM methods that significantly improved EEG/MEG localization from deep sources. In this study, we used the depth-weighted wMEM proposed in Afnan et al. (2024). Although depth weighting in the source imaging methods can improve localization accuracy from deep generators (Lin et al., 2006), these are often associated with large localization errors compared with superficial sources (Pascarella et al., 2023; Unnwongse et al., 2023). Interestingly, we found that the decrease in correlations between MEG and iEEG for deep ROIs was more pronounced in phase-based metrics than in amplitude-based metrics. This could be because AEC is estimated from the signal envelope, which is associated with synchronization over a larger scale, resulting in a higher SNR and reduced sensitivity to noise (Brookes et al., 2011a). *PLV* and wPLI*, on the other hand, rely on instantaneous phases and are linked to local synchrony. Phase-based measures could be more sensitive to noise and would therefore be more difficult to estimate from resting-state and deep sources using EEG/MEG source imaging. The assessment of phase and amplitude-based connectivity for superficial versus deep sources could be investigated in future studies and was beyond the scope of the current study. For the deep ROIs estimated by wPLI*, the cross-modal correlations between MEG and iEEG were very low and not statistically significant in any frequency bands. The reason why wPLI* estimations from deep sources were more affected than *PLV* was not clear. We repeated this analysis using another source imaging method (minimum norm estimate) and found a similar trend (result not shown). Comparison with minimum norm estimate was to check whether the choice of the source imaging method, wMEM, had influenced the results.

### Limitations

One limitation of this study is that the connectome available from the iEEG atlas covered only 44% of the whole brain. Despite not encompassing the entire brain, this approach represents the best means available to validate EEG/MEG-derived connectomes. Further validation could be considered with simultaneous EEG/MEG and iEEG recordings, although this can only provide even more limited spatial coverage unless it can be done on a large number of subjects. Another limitation is that we utilized a regularization parameter in wMEM, which was optimized for source estimation. The spatial prior model used in wMEM initializes each parcel using the MNE energy of the sources and therefore would be influenced by minimum norm estimate (MNE) regularization (Afnan et al., 2023). Through extensive MEG simulations, Vallarino et al. (2023) demonstrated that the regularization optimal for MEG source estimation was suboptimal for connectivity estimation. They showed an increased risk of being affected by spurious connections when using the regularization optimized for source estimation. Their findings suggested the need for less regularization to mitigate false positives. It may be necessary to reduce the regularization to improve the estimation of connectivity, a topic we plan to explore in future research. Additionally, since the study was conducted using simplistic simulated data, investigating how regularization could impact connectivity measures in the context of our multimodal real MEG/iEEG data could be valuable.

Another limitation is that the iEEG atlas, which is developed using iEEG channels from healthy brain regions, is still derived from patients with epilepsy. Studies using fMRI, EEG/MEG, and iEEG suggest that seizures or interictal epileptic activity can affect brain network properties even in regions distant from the epileptic focus (Aydin et al., 2020; DeSalvo et al., 2014; Lee et al., 2018; Taylor et al., 2022; van Diessen et al., 2013). However, this limitation is unavoidable since iEEG data are never collected from healthy subjects. Also, while regions may exhibit abnormalities in iEEG, these are unlikely to be consistent across patients as these are caused by the specific epileptic focus. Increasing the number of subjects/patients in each ROI pair (as illustrated in Figure 8, Supporting Information Figures S7 and S8) could mitigate these effects, potentially explaining the increased cross-modal correlations between MEG and iEEG when averaging more subjects.

Our goal was to validate the assessment of functional connectivity of normal brain activity using healthy MEG data. Currently, the iEEG atlas of normal brain activity (i.e., using iEEG channels exhibiting no epileptic activity) provides the best available ground truth for such validation with global brain coverage. One could argue that a more comparable MEG cohort would be a group of patients with epilepsy, excluding pathological regions as done in the iEEG atlas. However, excluding pathological regions from MEG would imply that we already trust MEG source imaging to identify these regions, which would make our validation objective redundant. The ideal dataset for this validation would be to consider challenging simultaneous iEEG and MEG recordings (Pizzo et al., 2019), but from a large group of patients, allowing for whole-brain coverage. However, such data are not yet available.

Another limitation of this study is that the distribution of patients’ ages in the iEEG atlas (31 ± 10 years, range: 13–62 years) was wider than that of the MEG dataset (28.67 ± 4.13 years, range: 20–38 years). We also acknowledge that the M-female [F] ratio was balanced in the iEEG dataset (F: 56, M: 54), whereas there were more Fs than Ms in our MEG dataset (F: 35, M: 10). Age and sex could influence resting-state oscillation properties (Hoshi & Shigihara, 2020; Schäfer et al., 2014). However, these effects are small (Hoshi & Shigihara, 2020), and given that the healthy subjects covered the range between the 25th (25 years) and 75th (40 years) percentiles of the patients’ age distribution, we believe our results to be minimally biased by age. Moreover, the effects of M-F differences were found to be less pronounced in eyes-closed conditions compared with eyes-open conditions (Hoshi & Shigihara, 2020), as this was the case for our study. For the comparisons between metrics such as AEC versus OAEC and PLV versus wPLI, both metrics are likely to be influenced by the same factors (age/sex), so we believe this should not bias our comparisons between metrics at the group level.

## CONCLUSIONS

This is the first validation of the MEG-derived connectome with the iEEG connectome at a group level. Based on the moderate spatial correlations between the two modalities, we can conclude that they share some commonalities. Differences in correlations estimated from different metrics may suggest that these metrics capture different/complementary aspects of brain activity. Moderate correlations were found between MEG and iEEG connectomes for metrics that include zero-lag connectivity. For metrics that removed or corrected zero-lag connectivity, the cross-modal correlations between MEG and iEEG decreased. This suggests that although correction of zero-lag connections may help in removing false connectivity related to volume conduction, it also removes true connections, as reflected in the overall decrease in cross-modal correlation between MEG and iEEG. In addition, a higher prevalence of zero-lag connectivity in MEG compared with iEEG was quantitatively presented.

## ACKNOWLEDGMENTS

This work was supported by Natural Sciences and Engineering Research Council of Canada (NSERC) Discovery grant, grant from Canadian Institutes of Health Research (CIHR; PJT-159448 and FDN 143208), and the Fonds de recherche du Québec—Nature et technologies (FRQNT) Research team grant. J.A. was partially supported by The Canadian Open Neuroscience Platform scholarship, Irma Bauer Fellowship, Faculty of Medicine and Health Sciences, McGill University, and Fonds de Recherche du Québec—Santé Doctoral scholarship. G.P. and A.G. were supported by the Italian Ministry of Health (GR-2019-12368960 and GR-2018-12366092). B.F. was supported by a CIHR project grant (PJT-175056), salary award (Chercheur-boursier clinicien Senior) of the Fonds de Recherche du Québec – Santé.

## SUPPORTING INFORMATION

Supporting information for this article is available at https://doi.org/10.1162/netn_a_00441.

## AUTHOR CONTRIBUTIONS

Jawata Afnan: Formal analysis; Investigation; Methodology; Writing – original draft; Writing – review & editing. Zhengchen Cai: Investigation; Methodology; Visualization; Writing – review & editing. Jean-Marc Lina: Investigation; Methodology; Writing – review & editing. Chifaou Abdallah: Investigation. Giovanni Pellegrino: Data curation. Giorgio Arcara: Data curation. Hassan Khajehpour: Methodology. Birgit Frauscher: Resources; Writing – review & editing. Jean Gotman: Conceptualization; Methodology; Supervision; Writing – review & editing. Christophe Grova: Conceptualization; Methodology; Supervision; Writing – review & editing.

## FUNDING INFORMATION

Jawata Afnan, Fonds de Recherche du Québec – Santé (https://dx.doi.org/10.13039/501100000156). Christophe Grova, CIHR, Award ID: PJT-159448. Jean Gotman, CIHR, Award ID: FDN 143208.

## Supplementary Material



## TECHNICAL TERMS

Resting-state brain: Brain activity at rest (awake; not engaged in a specific task or external stimuli).
Functional connectivity: Temporal relationship (e.g., correlation) between distinct brain regions measured using EEG/MEG, fMRI, or intracranial EEG.
Source leakage: The smearing of estimated brain generators around the true generators due to the volume conduction (EEG) or field spread (MEG).
Source imaging: The inverse problem allowing to estimate brain generators from sensor recordings (EEG/MEG).
Amplitude envelope correlation (AEC): A functional connectivity metric measuring the correlation between amplitude envelopes of two signals from different brain regions.
Phase locking value (PLV): A functional connectivity metric calculating the stability of phase difference between two signals over time (or trials).
Weighted phase lag index (wPLI): A metric assessing the consistency of phase lags between two signals, weighted by signals’ amplitudes and removing zero-lag connectivity.
Zero-lag connectivity: Synchronization between brain regions without any time delay, caused by true neuronal activity or source leakage.
Orthogonalized amplitude envelope correlation (OAEC): Modified AEC that addresses source leakage by orthogonalizing signals before calculating correlation.
