# Supplementary material for: Validating MEG estimated resting-state connectome with intracranial EEG
Source: Netw Neurosci. 2025 Mar 20;9(1):421–46. doi: 10.1162/netn_a_00441 (PMC11949576; doi:10.1162/netn_a_00441)
Supplement: Supplementary file 1 [file netn-9-1-421-s001.pdf]

Validating MEG estimated resting state connectome with intracranial EEG

Jawata Afnan, Zhengchen Cai, Jean Marc Lina, Chifaou Abdallah, Giovanni Pellegrino, Giorgio Arcara, Hassan Khajehpour, Birgit Frauscher, Jean Gotman & Christophe Grova

S1 Estimation of virtual iEEG (ViEEG) data from the MEG source map

We converted MEG-reconstructed source maps into iEEG channel space, by estimating corresponding iEEG potentials that would correspond to those MEG sources on each electrode contact (channel) of the atlas (Abdallah et al., 2022; Grova et al., 2016). To do so, we first localized the position of all channels of the atlas within the native MRI referential system of all healthy subjects from whom we analyzed MEG data. Co-registration between anatomical MRI of each subject and the ICBM152 template where the atlas is defined was obtained using Minctracc program (Collins et al., 1994). This is obtained in three steps: (1) estimation of a linear registration to account for the linear part of the transformation (using bestlinreg_s tool), (2) estimation of a non-linear transformation to account for the variability between the two maps (using minctracc tool); (3) application of the resulting non-linear transformation to the coordinates of the electrode contacts of MNI iEEG atlas, to convert them from the ICBM152 anatomy to the anatomy of each healthy subject.

Then, for each subject, to estimate the virtual iEEG potentials from MEG-estimated current density, J_{MSI}, we calculated a subject specific iEEG forward model, G_{iEEG} that estimates the influence of each dipolar source of the cortical surface on each iEEG channel (Grova et al., 2016). Since we did not intend to solve the inverse problem of source localization from iEEG data, we used a simplified iEEG forward model G_{iEEG} assuming an infinite volume conductor characterized by a conductivity σ of 0.25 S.m^{-1}. For a total number of iEEG contacts c, (c = 2300) and n number

of cortical sources (n = 8000). G_{iEEG} is a c x n matrix that estimates the electrical potential located at each iEEG electrode i (i =1, 2 …c) corresponding to an equivalent current dipole of unit activity located on the vertex S_j and oriented along $\vec{n}_j$, normal to the cortical surface (j = $1,2,\ldots n$), calculated as:

$$G_{iEEG}(i,j) = \frac{\vec{n}_j.\vec{u}_{ij}}{4\pi\sigma r_{ij}^2} \qquad \text{(S1)}$$

where $\vec{u}_{ij}$ is a unit vector oriented from the source S_j to the iEEG contact i and r_{ij} is the Euclidean distance between S_j and contact i. To avoid numerical instabilities, when the sources on the cortical surface were too close to the iEEG contacts (r_{ij} < 3 mm), the distance r_{ij} was set to 3 mm instead, keeping the orientation $\vec{u}_{ij}$. Finally, we applied the iEEG forward model, G_{iEEG} to the MEG-reconstructed source map (J_{MSI}) to estimate iEEG potentials on each iEEG channel, ViEEG as:

$$ViEEG = G_{iEEG} J_{MSI} \qquad \text{(S2)}$$

Here, we applied a simplified iEEG forward model because Cosandier-Rimélé et al. (2007) showed that it could estimate accurately real iEEG measurements. Moreover, von Ellenrieder et al. (2012) showed that the use of finite-element models considering the actual size and the shape of the iEEG electrodes had almost no influence on local electrical potentials at 2 mm from the electrodes.

S2 Bootstrapping iEEG data to investigate the spatial correlation between MEG and iEEG

The number of concatenated segments differed between the two modalities. For iEEG, data were visually selected as 60-second sections (either continuous or consecutive discontinuous 5-second segments after artifact exclusion) (Frauscher et al., 2018). For MEG, a 60-second segment was extracted for each subject, either continuous or concatenated, with a minimum continuous segment length of 10 seconds. Therefore, our selected data were composed of 12 or 6 concatenation points

at maximum, over the 1200 samples collected during 60s at 200Hz. To test whether this difference in concatenation affected the spatial correlation between the two modalities, we conducted a supplementary analysis by bootstrapping the iEEG data. We divided the 60 seconds of iEEG data into ten 6-second segments and performed bootstrapping with replacement 100 times. For each of the 100 bootstrapped iEEG datasets, we repeated the computations from Figures 3A and 3B by: (i) generating 5000 bootstrap resamples of the MEG connectome to reproduce the same subject/channel coverage as in the original iEEG data (see Section 2.3.4), and (ii) computing the correlation between iEEG and MEG connectomes for each bootstrap resample (Section 2.4). The results from the 100 bootstrapped iEEG datasets are shown in Fig. S2. The patterns for AEC and OAEC were similar to those illustrated in Figure 3B, demonstrating that the study's findings were consistent to other selections of iEEG signal and even when the data from the two modalities were concatenated from different segments.

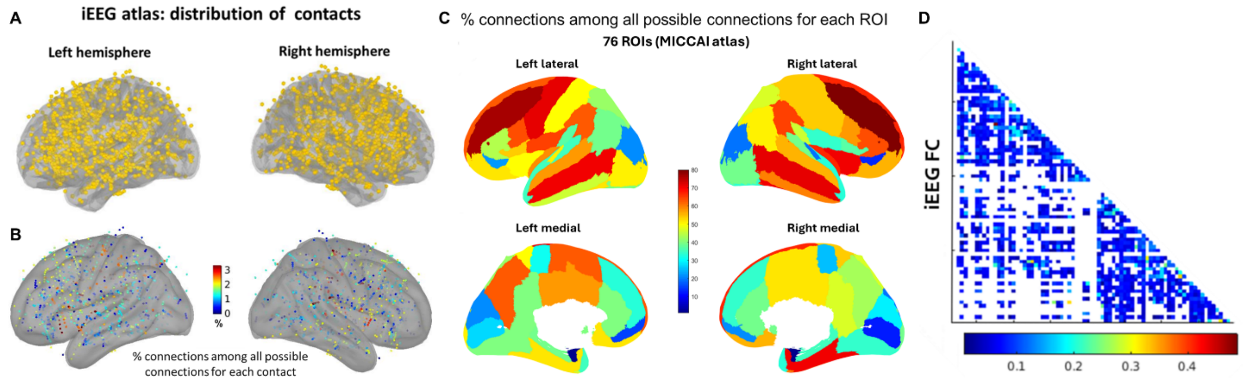

Fig S1: (A) Distribution of all iEEG contacts in the left and right hemispheres . (B) A subset of all iEEG contacts for which connectivity between two ROIs is available from the same patient is selected. The colormap in (B) shows the percentage of connections available for each contact among all possible connections for that contact to create the full connectome. (C) All contact pairs between ROIs are averaged to obtain a single value for each ROI pair. The colormap in (C) shows the percentage of connections available for each ROI among all possible connections to create the full connectome. (D) The resulting connectome covered 44% of the whole brain connectome, 62% of the left hemisphere connectome, 59% of the right hemisphere and 28% of interhemispheric connectome .

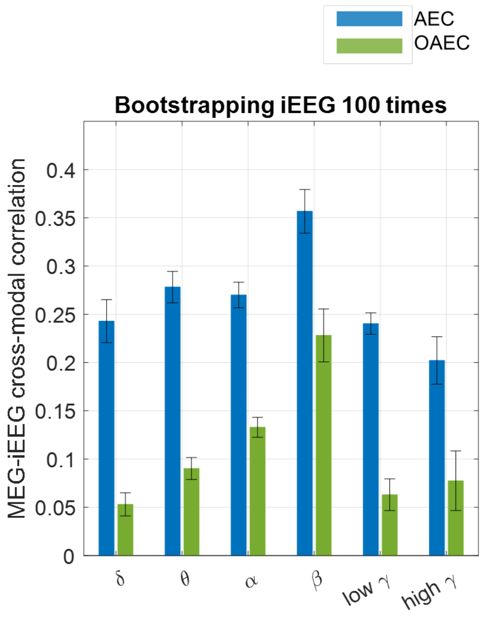

Fig S2: The median ± median absolute deviation of the 100 distributions of cross-modal correlations between MEG and iEEG (for 100 bootstrapped iEEG datasets). The iEEG data were divided into ten 6-second segments from a 60-second segment and bootstrapped 100 times with replacements from these segments. Results are shown for AEC and orthogonalized AEC.

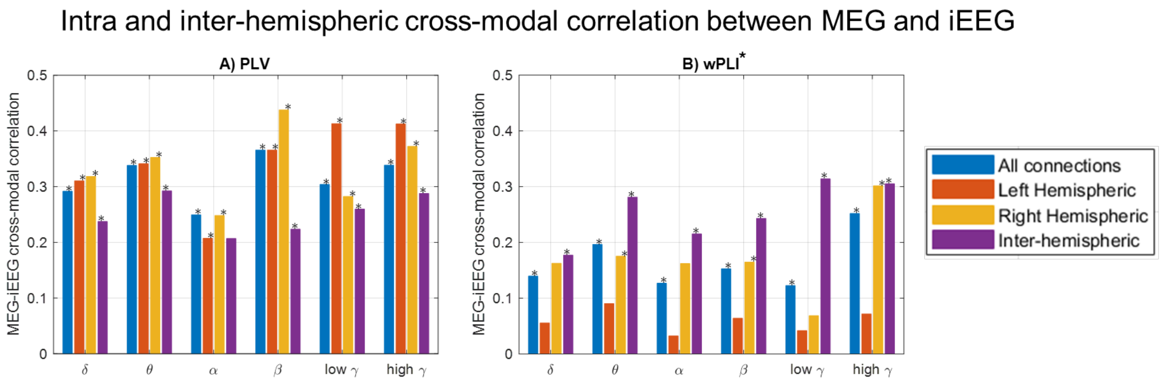

Fig S3: The median of the distribution of cross-modal correlations is depicted, considering all connections, intra-hemispheric connections, and inter-hemispheric connections for PLV (A) and wPLI* (B). The correlation was considered significant if its overlap with the null range was less than 2.5% (equivalent to a 5% two-tailed threshold, with 2.5% in each tail). Frequency bands with significantly higher correlations compared to the null distribution are marked with an asterisk (*).

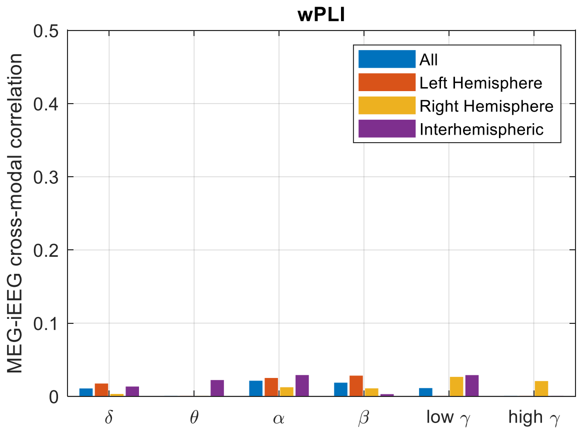

Fig S4: The median of the distribution of cross-modal correlations is depicted, considering all connections, intra-hemispheric connections, and inter-hemispheric connections for wPLI calculated using the original definition proposed in Vinck et al. (2011) (i.e. when including envelope amplitudes). The correlation was considered significant if its overlap with the null range was less than 2.5% (equivalent to a 5% two-tailed threshold, with 2.5% in each tail). Frequency

bands with significantly higher correlations than the null distribution are marked with an asterisk (*).

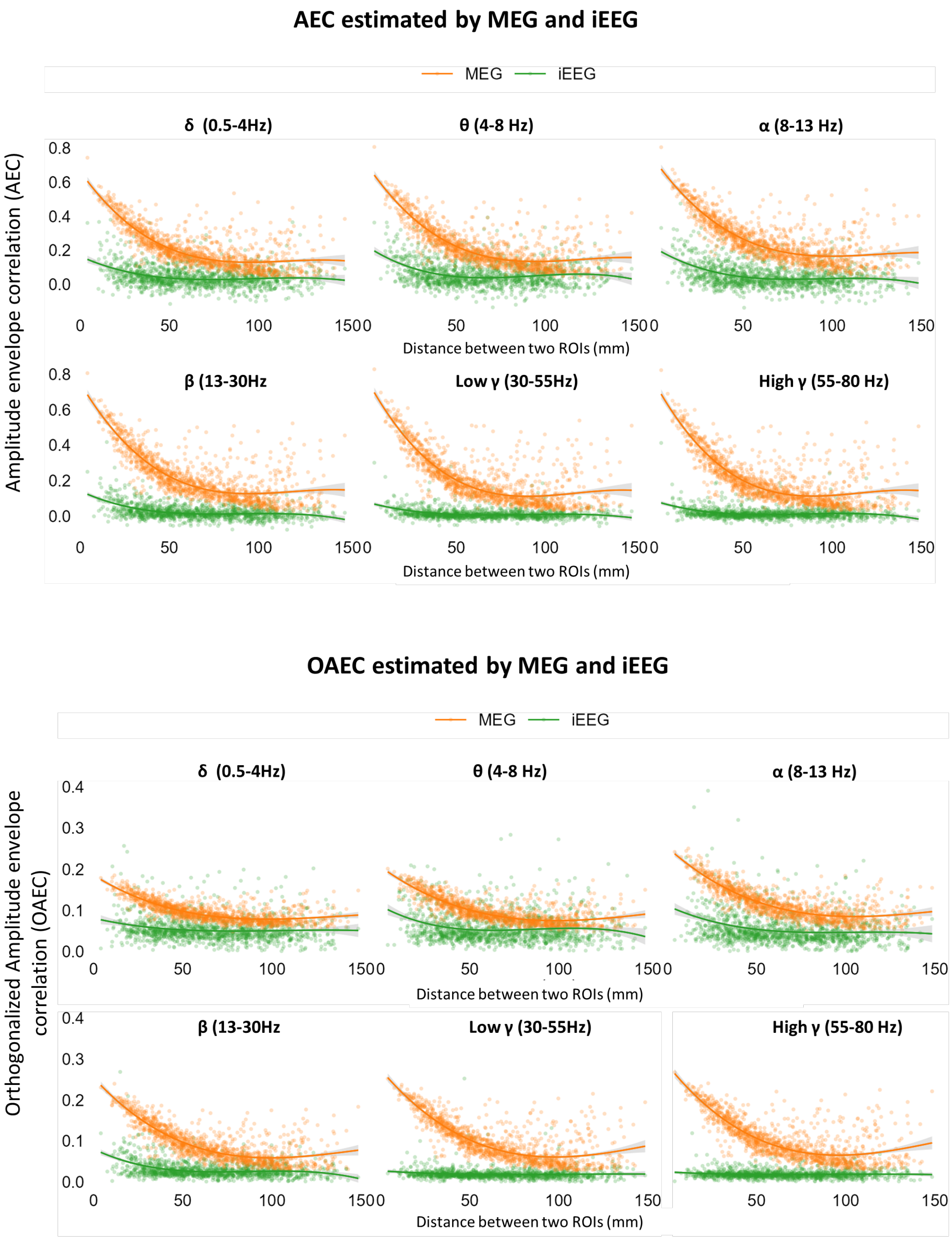

Fig S5: AEC and OAEC for MEG and iEEG for different frequency bands as a function of the distance between two ROIs.

Difference of OAEC and AEC for MEG and iEEG

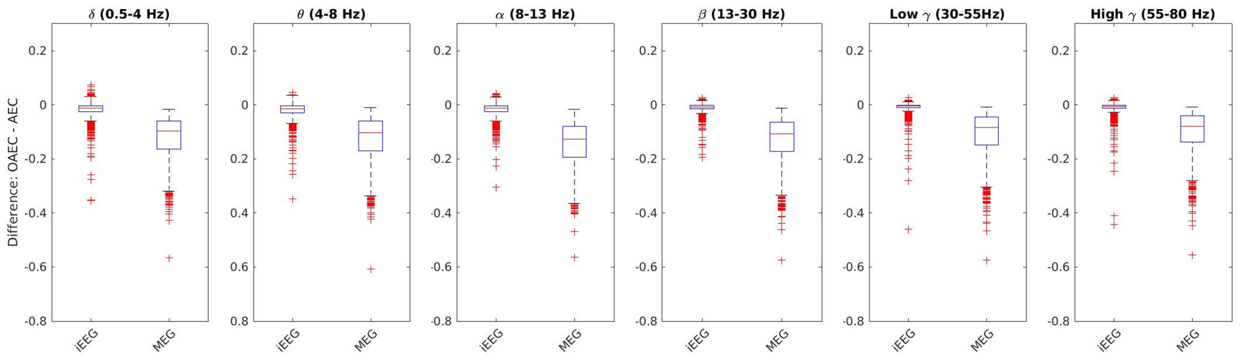

Fig S6: The difference between OAEC and AEC (OAEC minus AEC) for MEG and iEEG for all frequency bands.

PLV estimated by MEG and iEEG

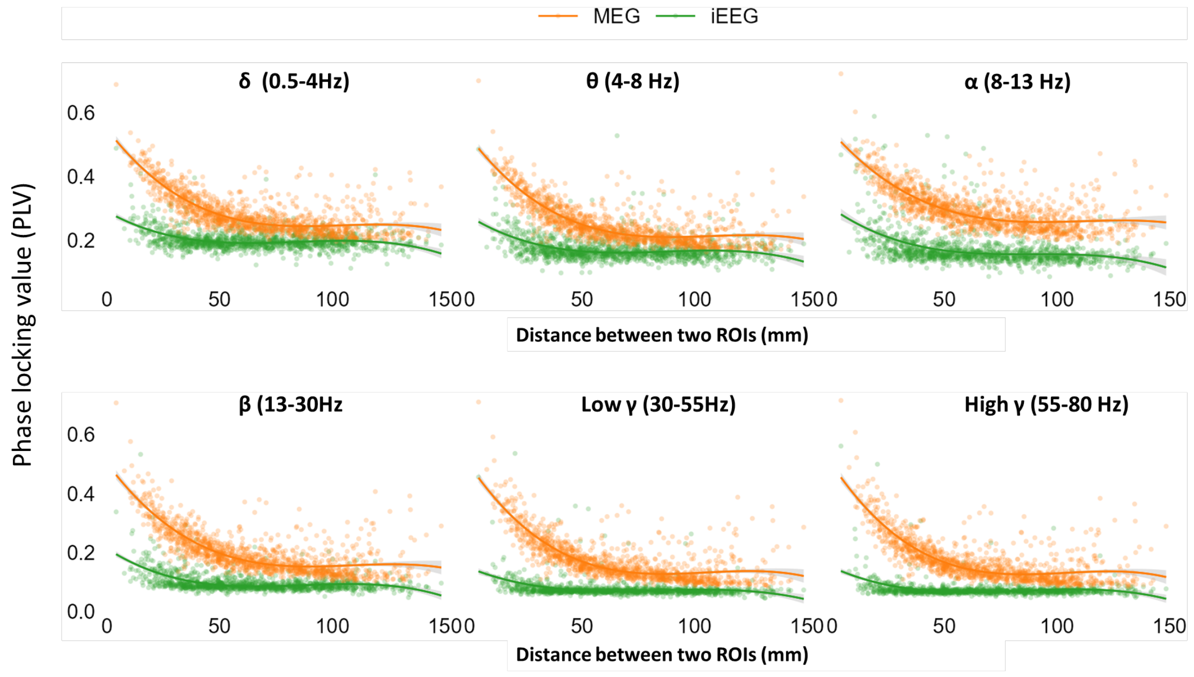

wPLI* estimated by MEG and iEEG

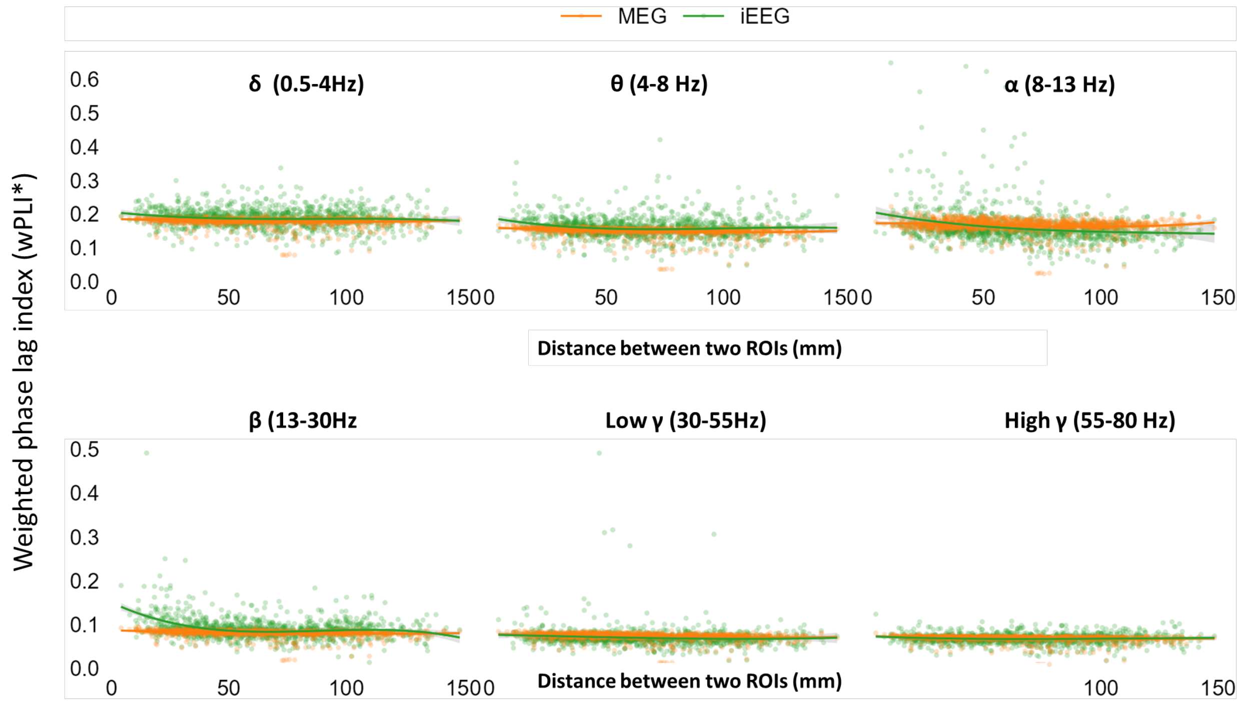

Fig S7: PLV and wPLI* for MEG and iEEG for different frequency bands as a function of the distance between two ROIs.

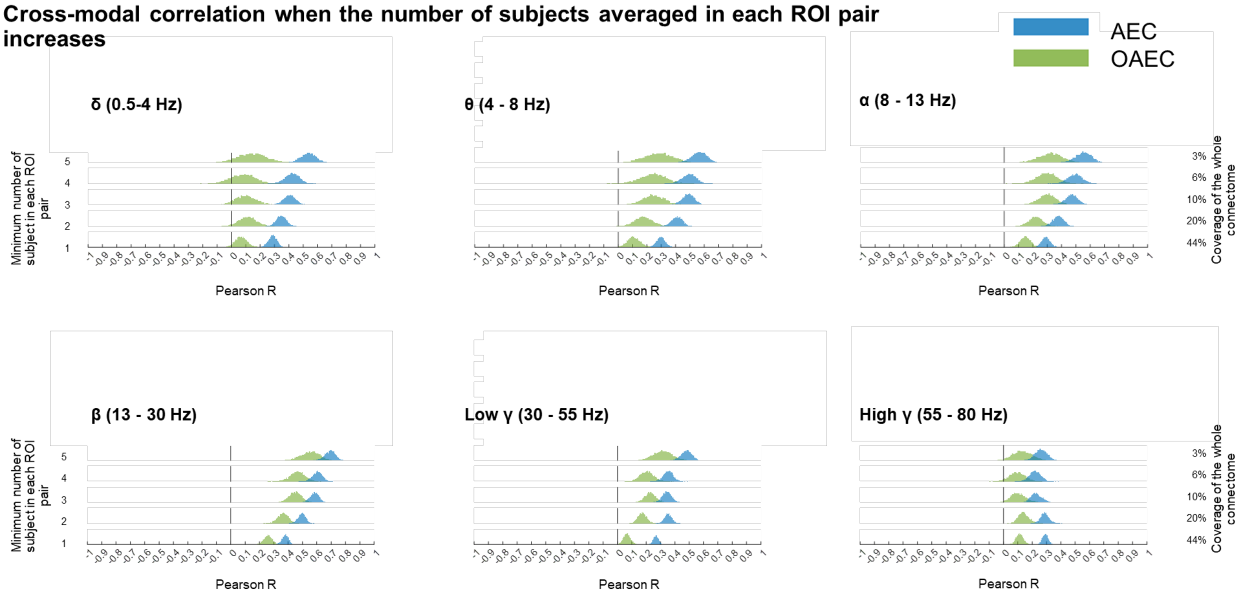

Fig S8: The cross-modal spatial correlations between MEG and iEEG connectomes obtained using AEC and $OAEC$ as we increase the minimum number of subjects from 1 to 5 in each ROI pair. Increasing the minimum number of subjects in each ROI pair (as shown on the left) decreases the available coverage of the iEEG connectome from 44% to 3% (as shown on the right). For example, the bottom row displays histograms of the correlations between MEG and iEEG connectomes when the iEEG connectome was created with ROI pairs that include at least 1 patient, covering 44% of the connectome.

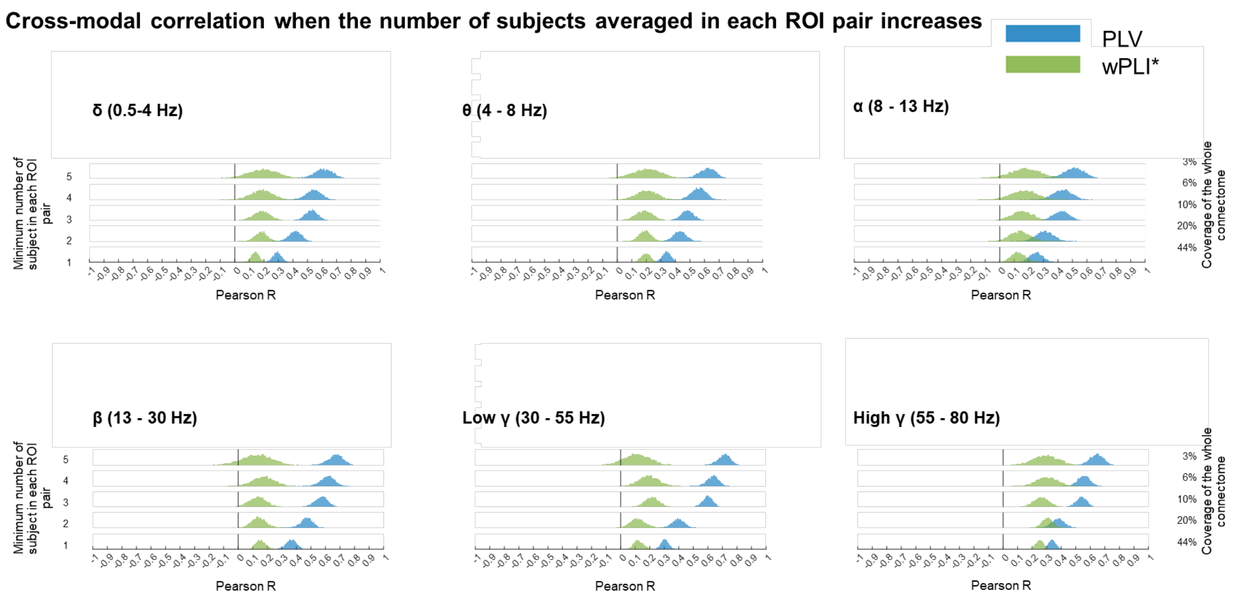

Fig S9: The cross-modal spatial correlations between MEG and iEEG connectomes obtained using PLV and $wPLI^*$ as we increase the minimum number of subjects from 1 to 5 in each ROI pair. Increasing the minimum number of subjects in each ROI pair (as shown on the left) decreases the available coverage of the iEEG connectome from 44% to 3% (as shown on the right). For example, the bottom row displays histograms of the correlations between MEG and iEEG connectomes when the iEEG connectome was created with ROI pairs that include at least 1 patient, covering 44% of the connectome.

References:

Abdallah, C., Hedrich, T., Koupparis, A., Afnan, J., Hall, J. A., Gotman, J., Dubeau, F., von Ellenrieder, N., Frauscher, B., & Kobayashi, E. (2022). Clinical Yield of Electromagnetic Source Imaging and Hemodynamic Responses in Epilepsy: Validation With Intracerebral Data. *Neurology*.

Collins, D. L., Neelin, P., Peters, T. M., & Evans, A. C. (1994). Automatic 3D intersubject registration of MR volumetric data in standardized Talairach space. *Journal of computer assisted tomography*, *18*(2), 192-205.

Cosandier-Rimélé, D., Badier, J.-M., Chauvel, P., & Wendling, F. (2007). A physiologically plausible spatio-temporal model for EEG signals recorded with intracerebral electrodes in human partial epilepsy. *IEEE transactions on biomedical engineering*, *54*(3), 380-388.

Grova, C., Aiguabella, M., Zelmann, R., Lina, J. M., Hall, J. A., & Kobayashi, E. (2016). Intracranial EEG potentials estimated from MEG sources: A new approach to correlate MEG and iEEG data in epilepsy. *Human brain mapping*, *37*(5), 1661-1683.

Vinck, M., Oostenveld, R., Van Wingerden, M., Battaglia, F., & Pennartz, C. M. (2011). An improved index of phase-synchronization for electrophysiological data in the presence of volume-conduction, noise and sample-size bias. *NeuroImage*, *55*(4), 1548-1565.

von Ellenrieder, N., Beltrachini, L., & Muravchik, C. H. (2012). Electrode and brain modeling in stereo-EEG. *Clinical Neurophysiology*, *123*(9), 1745-1754.
